# Combinatorial therapy regimens targeting preclinical models of melanoma resistant to immune checkpoint blockade

**DOI:** 10.1172/JCI185220

**Published:** 2025-07-10

**Authors:** Imran Khan, Aida Rodriguez-Brotons, Anukana Bhattacharjee, Vladimir Bezrookove, Altaf Dar, David De Semir, Mehdi Nosrati, Ryan Ice, Liliana Soroceanu, Stanley P. Leong, Kevin B. Kim, Yihui Shi, James E. Cleaver, James R. Miller, Pierre-Yves Desprez, John M. Kirkwood, Marcus Bosenberg, Nathan Salomonis, Sean McAllister, Mohammed Kashani-Sabet

**Affiliations:** 1 California Pacific Medical Center (CPMC) Research Institute, San Francisco, California, USA.; 2 Center for Melanoma Research and Treatment, CPMC, San Francisco, California, USA.; 3 Division of Biomedical Informatics, Cincinnati Children’s Hospital Medical Center, Cincinnati, Ohio, USA.; 4 Department of Dermatology, University of California San Francisco, San Francisco, California, USA.; 5 Melanoma Program, Division of Medical Oncology, Department of Medicine, UPMC Hillman Cancer Center, Pittsburgh, Pennsylvania, USA.; 6 Department of Dermatology, Yale University, New Haven, Connecticut, USA.

**Keywords:** Dermatology, Oncology, Cancer immunotherapy, Expression profiling, Melanoma

## Abstract

Few effective therapeutic options exist after progression on immune checkpoint blockade (ICB) for melanoma. Here, we utilized a platform incorporating transcriptomic profiling, high-throughput drug screening, and murine models to demonstrate the preclinical efficacy of several combinatorial regimens against ICB-resistant melanoma. Transcriptomic analysis of ICB-resistant melanomas demonstrated activation of several targetable pathways. High-throughput drug screening targeting these pathways identified several effective combinations in ICB-resistant patient-derived xenograft models. The combination of cobimetinib and regorafenib (termed Cobi+Reg) emerged as a particularly promising regimen, with efficacy against distinct molecular melanoma subtypes and after progression on ICB in immunocompetent models. Transcriptomic and spatial analysis of Cobi+Reg–treated tumors demonstrated upregulation of antigen presentation machinery, with concomitantly increased activated T cell infiltration. Combining Cobi+Reg with ICB was superior to either modality in vivo. This analytical platform exploits the biology of ICB-resistant melanoma to identify therapeutic vulnerabilities, resulting in the identification of drug combinations that form the basis for rational clinical trial design in the setting of advanced melanoma resistant to ICB.

## Abstract 2

This study leverages the biology of melanoma resistant to immune checkpoint blockade to identify effective combinatorial therapies that form the basis for rational clinical trial design for this treatment-refractory patient population.

## Graphical abstract

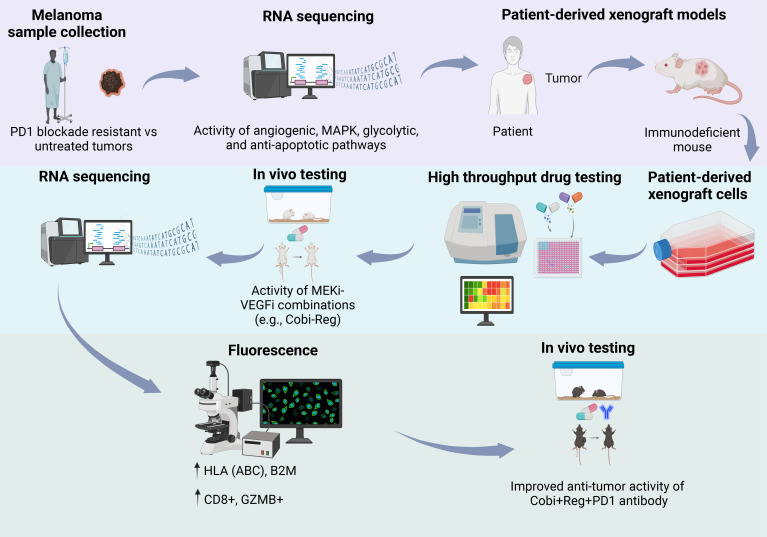

## Introduction

Melanoma is an important clinical problem, as the fifth most common malignancy in the United States (1). The development of targeted agents and immunotherapies has revolutionized the management of advanced melanoma. Immune checkpoint blockade (ICB) has emerged as a mainstay of melanoma therapy, using PD-1 (2, 3) and CTLA-4 (4) blockade alone or in combination (5). Recently, the combination of PD-1 and LAG-3 blockade has also shown clinical benefit (6). These immunological approaches have been extended to the adjuvant setting, with FDA approval of single-agent nivolumab (7) and pembrolizumab (8) for high-risk, resected node-positive and node-negative melanoma. Despite these advances, a substantial proportion of patients with advanced melanoma exhibit either intrinsic or acquired resistance to first-line therapies. Once resistance occurs, treatment options are extremely limited, highlighting the urgent need to identify effective therapies for patients progressing on ICB. Given the highly refractory nature of this patient population, combinatorial therapies will likely be required to successfully treat melanoma in this setting.

Numerous studies have been performed to identify pretreatment biomarkers of response or resistance to ICB (9–12). Although concordance between these studies is frequently lacking, some common themes have emerged, with PD-L1 expression level (9), an inflamed tumor microenvironment (10, 11), and high tumor mutational burden (12) each predicting a higher level of response to ICB. In addition, numerous investigations have been conducted into mechanisms of resistance to ICB (reviewed in refs. 13, 14). Recurring themes include changes in tumor microenvironment and neovasculature, as well as tumor immunorecognition; antigen presentation, including components of the MHC; neoantigen repertoire; and T cell repertoire. Prominent efforts are underway to identify immunological interventions that may resensitize melanoma to ICB. However, to date, relatively little attention has been paid to exploit the tumor-intrinsic biology of ICB-resistant melanoma to develop therapies to specifically target this treatment-refractory patient population. In this study, we assessed the transcriptomic profiles of ICB-resistant melanoma and identified several targetable genes and pathways, resulting in the development of an effective combinatorial therapeutic approach validated in multiple in vivo models of ICB resistance.

## Results

### Transcriptomic analysis of ICB-resistant melanoma.

**Figure 1:**
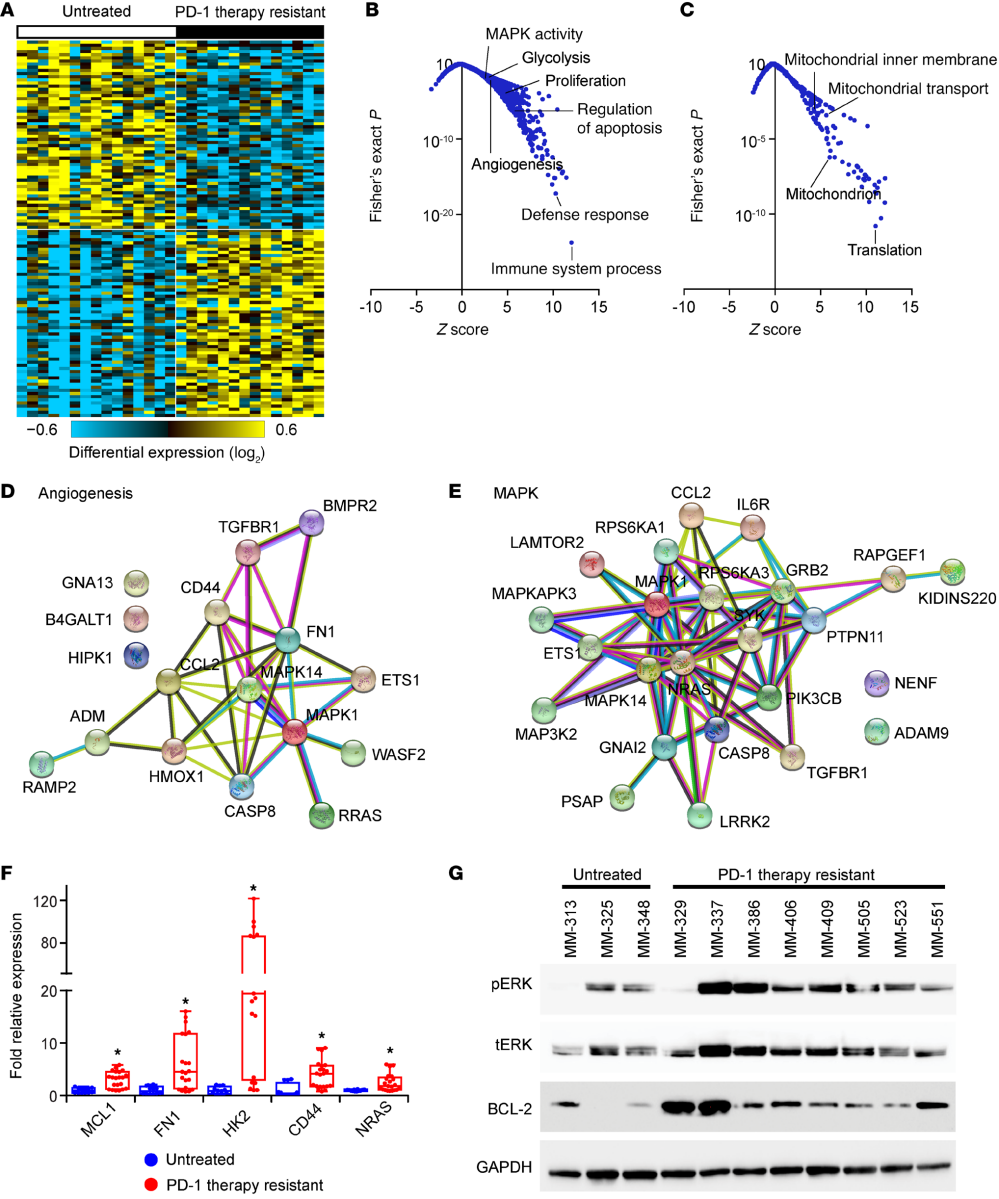
Identification of differentially expressed genes and pathways in ICB-resistant melanoma. (**A**) Heatmap of supervised analysis of RNA-Seq results from untreated metastatic melanoma specimens versus tumors obtained from patients progressing on PD-1 blockade. The *z* scores of upregulated (**B**) and downregulated (**C**) biological processes (as determined by Fisher’s exact test) identified by Gene Ontology analysis. (**D** and **E**) WikiPathways analysis of differentially expressed genes in ICB-resistant melanomas involved in angiogenesis (**D**) and MAPK pathway (**E**). (**F**) qRT-PCR analysis of expression of various differentially expressed genes in ICB-resistant patient samples; *P < 0.05 by Student’s *t* test. (**G**) Western blot analysis of expression of various proteins in pathways identified by RNA-Seq analysis.

Initially, we aimed to comprehend unique features of the biology of ICB-resistant melanoma in our own patient population. To this end, we performed bulk RNA-Seq analysis comparing 14 metastatic melanoma tumors from patients whose disease progressed after PD-1 blockade versus 15 tumors from treatment-naive patients with metastatic melanoma (Supplemental Table 1; supplemental material available online with this article; https://doi.org/10.1172/JCI185220DS1). Supervised hierarchical analysis identified 516 differentially overexpressed and 139 downregulated genes in ICB-resistant melanoma (Figure 1A and Supplemental Table 2). Gene Ontology analysis identified numerous differentially affected biological processes, including upregulation of cell proliferation, angiogenesis, MAPK, glycolysis, and regulation of apoptosis (Figure 1B), along with downregulation of the mitochondrial protein complex and respiratory electron transport chain (Figure 1C). Specifically, RNA-Seq analysis identified dysregulation of multiple genes in these signaling pathways that could provide druggable opportunities, including upregulation of genes involved in angiogenic (e.g., *FN1* and *CD44*), MAPK (e.g., *NRAS* and *MAPK1*), glycolytic (e.g., *HK2* and *PGK1*), and antiapoptotic pathways (e.g., *MCL1* and *TNFRSF1B*) (Supplemental Table 2). In addition, the downregulation of several genes involved in mitochondrial function (e.g., *NDUFA3* and *NDUFB1*) (Supplemental Table 2) was of interest, as this downregulation has been shown to activate multiple retrograde signaling pathways, including MAPK and phosphoinositide 3 kinase, ultimately resulting in increased levels of BCL2-family proteins and promoting resistance to apoptotic stimuli (15–18). We further assessed the contribution of the identified differentially expressed genes to the pathways identified by Gene Ontology analysis using WikiPathways, focusing on the angiogenic (Figure 1D), MAPK (Figure 1E), glucose metabolism (Supplemental Figure 1A), mitochondrial (Supplemental Figure 1B), and apoptotic (Supplemental Figure 1C) gene signatures. Quantitative RT-PCR (qRT-PCR) analysis confirmed differential expression of several of these potentially actionable genes (Figure 1F). In addition, we cross-compared MAPK pathway signaling and BCL2 expression between the treatment-naive and treatment-resistant groups and observed marked overexpression in the resistant subset (Figure 1G), in agreement with RNA-Seq analysis.

### Pharmacological targeting of ICB-resistant melanoma.

**Figure 2:**
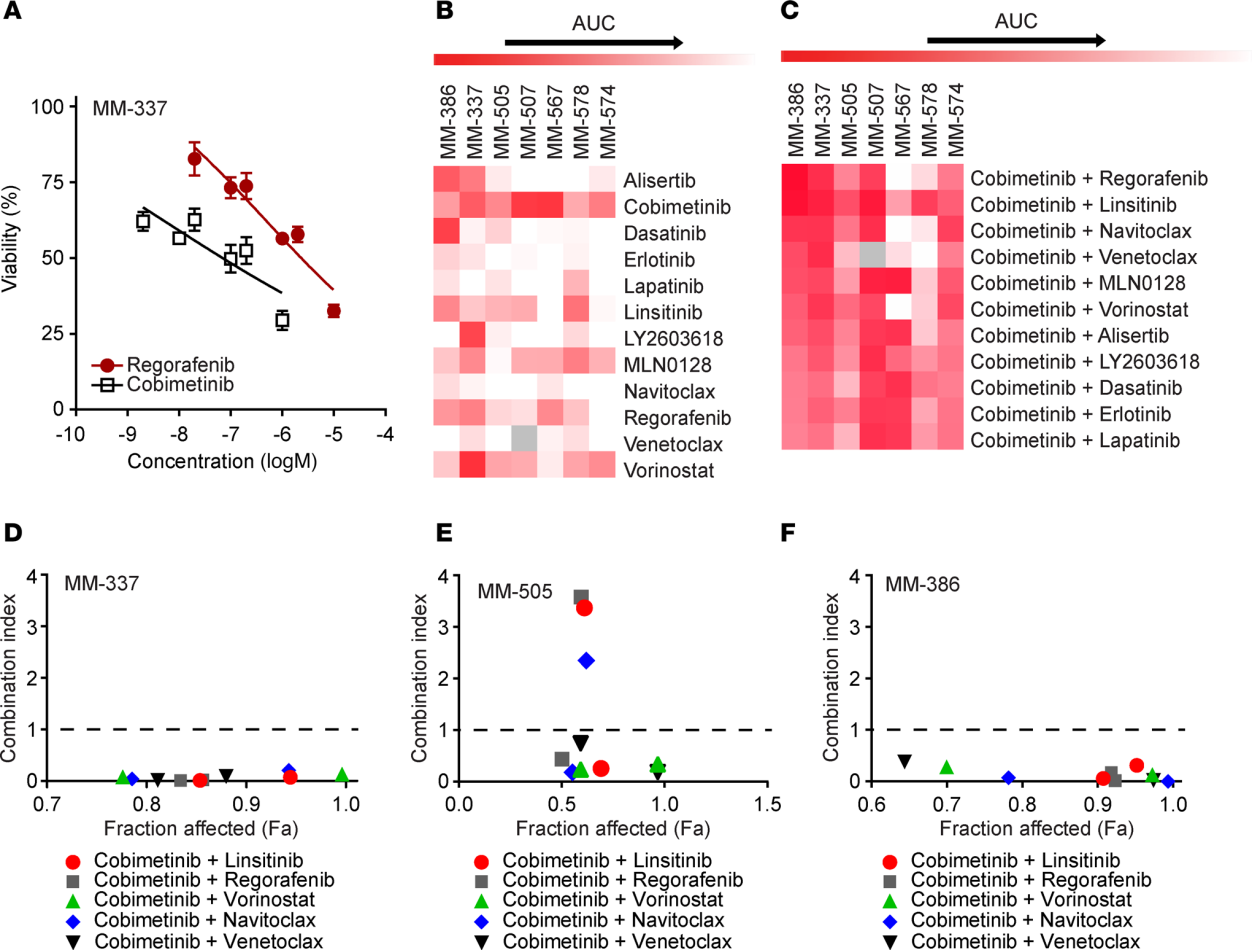
Identification of active drugs against ICB-resistant melanoma using high-throughput drug screening. (**A**) Representative 6-point concentration-response curves generated in MM-337 PDXC are shown for cobimetinib and regorafenib. Heatmap of high-throughput drug screening analysis demonstrating the effects on cell viability of the top drugs alone (**B**) and in combination (**C**) in treatment-resistant MM-386, MM-337, MM-505, MM-507, MM-567, MM-578, and MM-574 PDXCs. Percentage of cell viability was equal to Treatment_A_/Control_A_ × 100%, where A = absorbance. Darkest red of the heatmap indicates 0% cell viability/100% inhibition, whereas white indicates 100% cell viability/0% inhibition. (**D**–**F**) Combination index values for various drug combinations in (**D**) MM-337, (**E**) MM-505, and (**F**) MM-386 PDXCs. The fraction affected represents the percentage of cells killed (e.g., 0.2 = 20%) by each of the drug combinations evaluated.

In order to develop a therapeutic strategy to target ICB-resistant melanoma, we designed a custom high-throughput drug screening (HTDS) (Supplemental Table 3) focused on the RNA-Seq results as well as on classical therapeutic vulnerabilities previously described in melanoma (19–26). We included 21 drugs in our platform, including those targeting MAPK signaling (e.g., cobimetinib), glycolysis/IGF1R pathway (e.g., linsitinib), angiogenesis (e.g., regorafenib), and BCL2 (e.g., navitoclax or venetoclax). Short-term patient-derived xenograft (PDX) cultures (termed PDXCs) plated as tumor-spheres were treated with individual drugs and drug combinations, and cell viability was assessed. To evaluate drug interactions, a custom HTDS of multiple melanoma PDXCs was developed similar to that previously described by our group (27). A strategy of our HTDS platform was to set the highest concentration used in the assay to the C_max_ reported for each of the drugs in clinical trials (Supplemental Table 3). Concentration-response curves were run for each drug alone (with an example provided in Figure 2A; also see Supplemental Figure 2A and Supplemental Table 4). The drugs were then combined at 2 fixed ratios: C_max_ and 10% C_max_ for analysis of drug interactions, including 2 fixed ratios that control against false-positives and allow further ranking of drug effects to favor drug combinations that produce the greatest effects at the lowest concentration (10% C_max_) (Supplemental Figure 2B and Supplemental Table 5). The evaluation was performed using 7 PDXC melanoma models of PD-1 antibody resistance. The most effective drug combinations were ranked by their overall ability to decrease cell viability (as determined by the AUC) across all PDXCs at 10% C_max_. As an example, the activity of the top individual drugs (Figure 2B and Supplemental Table 6) and the most effective drug combinations identified (Figure 2C and Supplemental Table 7) are shown. Although administration of single drugs revealed modest effects on tumor cell viability (Figure 2B), combinatorial drug treatment identified numerous active combinations (Figure 2C). Several of the most effective drug combinations were further evaluated by performing a combination index analysis (28) using data obtained from the full concentration-response analysis of individual drugs and their response in the fixed ratios of the drug combinations. A combination index value of less than 1 indicates synergism, equal to 1 is additive effect, and greater than 1 is antagonism. As shown in Figure 2, D–F, these combinations showed synergistic interactions across several of the PDXCs evaluated. Importantly, many of the top effective drug combinations are in agreement with pathway vulnerabilities identified by RNA-Seq analysis.

### Antitumor activity of combinatorial drug therapy.

**Figure 3:**
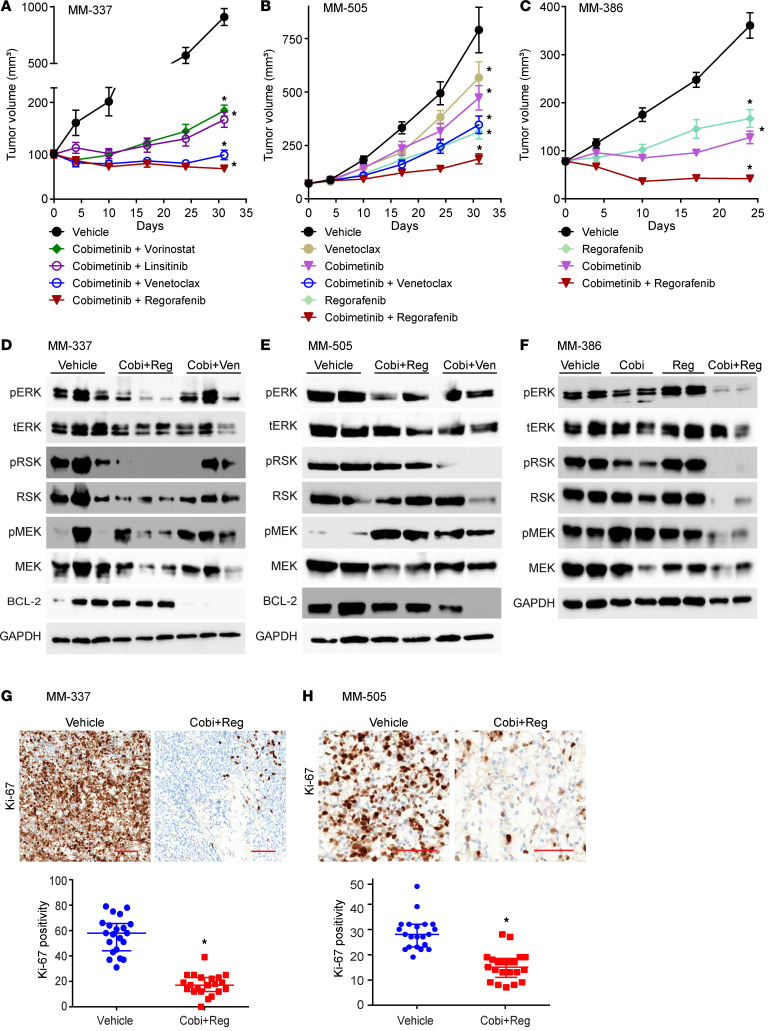
Effects of single or combination drug treatments in various melanoma PDX models. (**A**–**C**) Antitumor activity of various single drugs or drug combinations on the following PDX models in vivo, respectively: MM-337 (**A**), MM-505 (**B**), and MM-386 (**C**); *P < 0.05 using 2-way ANOVA repeated measures and a Tukey’s multiple-comparison test. (**D**–**F**) Western blot analysis of expression of various proteins in MM-337 (**D**), MM-505 (**E**), and MM-386 (**F**) in vivo tumors treated with vehicle or various drugs or drug combinations. Each column represents a tumor harvested from a different mouse in each treatment group. (**G** and **H**) Representative IHC images and quantification of Ki-67 staining of MM-337 (**G**) and MM-505 (**H**) in vivo tumors treated with vehicle or cobimetinib plus regorafenib; *P < 0.05 by Student’s *t* test. Scale bar: 100 μm.

Based on these results, 4 drug combinations were selected for in vivo determination of antitumor activity in MM-337 (Figure 3A), a *BRAF*-mutant PDX line developed after progression on combined ICB with anti–PD-1 and anti–CTLA-4 antibody (as well as BRAF and MEK inhibition) (Supplemental Table 1): cobimetinib plus regorafenib (Cobi+Reg), cobimetinib plus venetoclax (Cobi+Ven), cobimetinib plus linsitinib, and cobimetinib plus vorinostat. Although all 4 combinations produced statistically significant antitumor activity, 2 combinations (Cobi+Reg and Cobi+Ven) produced the greatest reduction in tumor volume, including evidence of tumor regression. The Cobi+Reg and Cobi+Ven regimens were then tested in MM-505, an *NF-1*–mutant PDX line developed after progression on PD-1 blockade. Both regimens produced statistically significant antitumor activity and were superior to each of the single agents alone. However, the Cobi+Reg combination was superior to Cobi+Ven in the MM-505 model (Figure 3B) and emerged as the lead candidate for further testing and characterization. Subsequently, Cobi+Reg was tested in the MM-386 model, an *NRAS*-mutant PDX line developed after progression on PD-1 blockade, and was superior to either agent alone, including evidence of tumor regression (Figure 3C), similar to that observed with the MM-337 model. Thus, Cobi+Reg produced marked antitumor efficacy in multiple PDX lines encompassing the major molecular subtypes of melanoma (i.e., *BRAF*-, *NRAS*-, and *NF-1*–mutant) developed after progression on PD-1–based ICB. We also tested the activity of Cobi+Reg in a panel of 5 ICB-naive human melanoma PDX lines in culture and in the MM-363 line in vivo. The results in culture demonstrated reduced antitumor activity of the combination in these treatment-naive models (Supplemental Figure 2C and Supplemental Table 8) when compared with the treatment-resistant models. In vivo testing revealed antitumor activity for Cobi+Reg in the MM-363 model (Supplemental Figure 2D).

**Figure 4:**
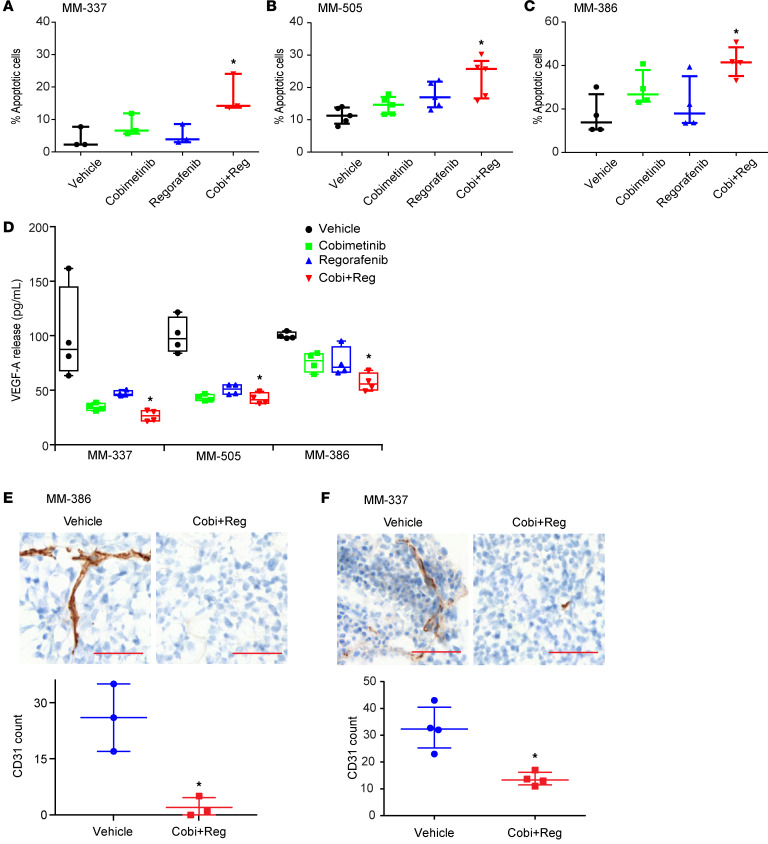
Effects of single or combination drug treatments on various cellular processes in distinct melanoma PDX models. (**A**–**C**) Effects of cobimetinib plus regorafenib treatment on apoptosis using caspase 3/7 analysis 48 hours after drug treatment in vitro of MM-337 (**A**), MM-505 (**B**), and MM-386 cells (**C**); *P < 0.05 by Student’s *t* test for vehicle versus drug combination. (**D**) Quantification of VEGF-A expression by ELISA 24 or 48 hours after drug treatment in MM-337, MM-505, or MM-386 cells; *P < 0.05 by Student’s *t* test for vehicle versus drug combination. (**E** and **F**) Representative IHC images and quantification of CD31 staining in MM-386 (**E**) and MM-337 (**F**) in vivo tumors treated with cobimetinib + regorafenib or vehicle; *P < 0.05 by 2-tailed unpaired Student’s *t* test. Scale bar: 50 μm.

We then evaluated whether effective combinatorial therapy affects the molecular profiles of ICB-resistant melanoma. There was a profound reduction in MAPK pathway activity (as evidenced by substantially reduced pERK and pRSK-90 protein levels) after Cobi+Reg and Cobi+Ven treatment in each of the 3 PDX lines tested (Figure 3, D–F, and Supplemental Figure 3, A–C), whereas Cobi+Ven administration also resulted in marked suppression of BCL2 expression (Figure 3, D and E). Accordingly, Cobi+Reg treatment produced a statistically significant reduction in proliferative capacity, as evidenced by suppressed Ki-67 immunostaining in vivo (Figure 3, G and H, and Supplemental Figure 3D). In addition, Cobi+Reg administration resulted in an increased apoptotic index, as assessed by caspase 3/7 levels (Figure 4, A–C, and Supplemental Figure 4, A–C). Finally, Cobi+Reg treatment resulted in statistically significantly reduced secretion of VEGFA in culture (Figure 4D), with concomitantly suppressed microvessel density in vivo (as evidenced by reduced CD31 immunostaining) (Figure 4, E and F, and Supplemental Figure 4D). Thus, administration of combinatorial therapy that was effective against ICB-resistant PDX models reversed key hallmarks of the biology of ICB-resistant melanoma observed in drug-resistant patient tumors.

**Figure 5:**
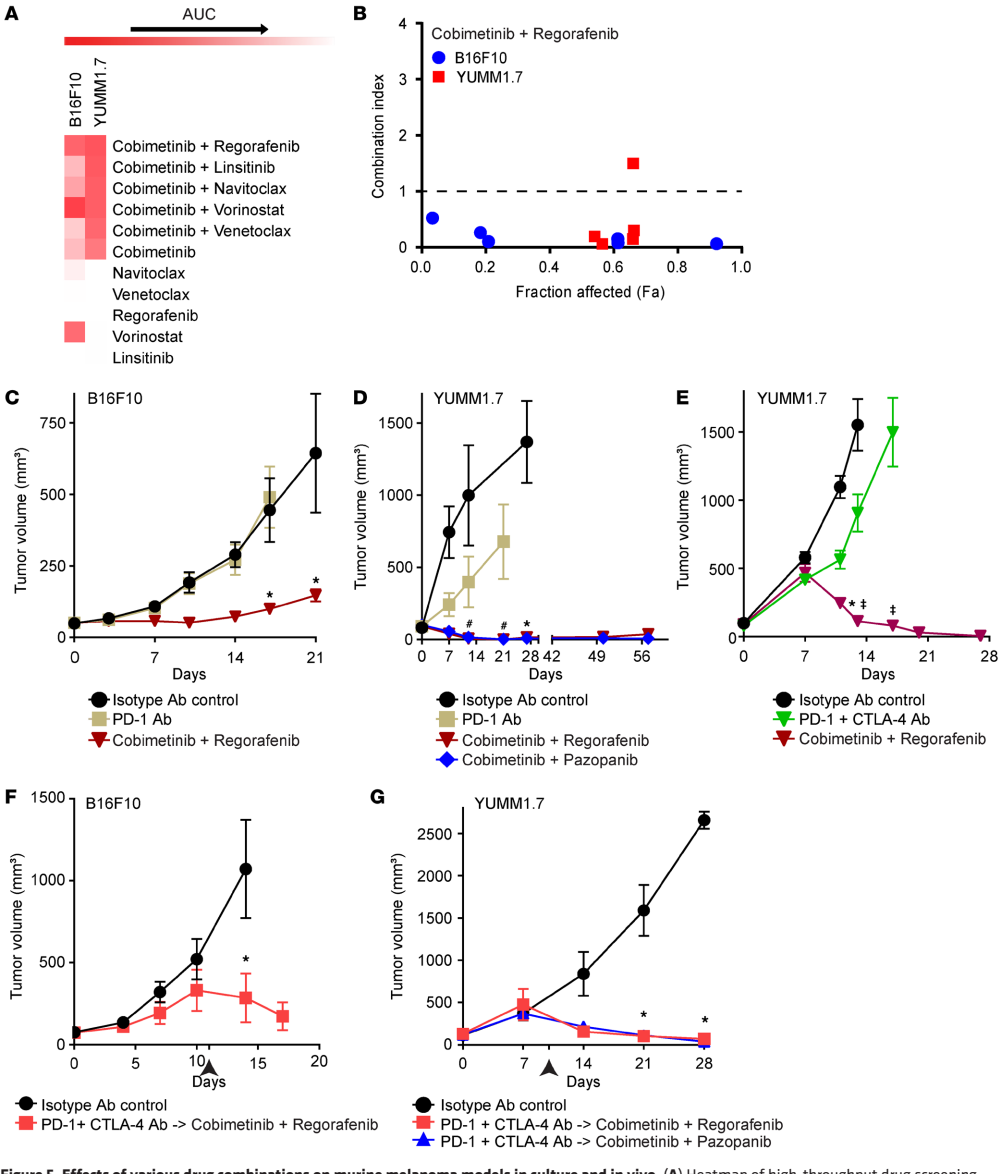
Effects of various drug combinations on murine melanoma models in culture and in vivo. (**A**) Heatmap of high-throughput drug screening analysis demonstrating effects on cell viability of the top single drugs and drug combinations in B16F10 and YUMM1.7 cells. (**B**) Combination index of cobimetinib plus regorafenib (Cobi+Reg) treatment in B16F10 and YUMM1.7 cells. The heatmap illustration and combination index value demonstration are similar to that described for Figure 2. (**C**) Antitumor activity of various drugs on B16F10 melanoma; *P < 0.05 by randomization test. (**D**) Antitumor activity of various drugs on YUMM1.7 melanoma; *P < 0.05 by randomization test for isotype versus Cobi+Reg or cobimetinib plus pazopanib (Cobi+Paz); #*P* < 0.05 by randomization test (with Bonferroni’s correction) for PD-1 antibody (Ab) versus Cobi+Reg or Cobi+Paz. (**E**) Antitumor activity of various drugs on YUMM1.7 melanoma; *P < 0.05 by Student’s *t* test for isotype versus Cobi+Reg (with Bonferroni’s correction); ‡*P* < 0.05 by Student’s *t* test (with Bonferroni’s correction) for PD-1+CTLA-4 Ab versus Cobi+Reg. (**F**) Antitumor activity of various drugs on B16F10 and (**G**) YUMM1.7 melanoma; *P < 0.05 by Student’s *t* test for isotype versus PD-1+CTLA-4 Ab-> Cobi+Reg or PD-1+CTLA-4 Ab-> Cobi+Paz (with Bonferroni’s correction) (**G**). The arrowhead represents the time point of treatment crossover.

Subsequently, we assessed the activity of Cobi+Reg (as well as other promising drug combinations) in the B16F10 and YUMM1.7 immunocompetent murine melanoma models, which have been shown to be refractory to ICB (29, 30). Drug treatment in culture showed synergistic activity for the Cobi+Reg combination in both cell lines (Figure 5, A and B, and Supplemental Table 9). In vivo testing showed evidence of marked antitumor activity for Cobi+Reg in the B16F10 model, which was superior to treatment with PD-1 blockade (Figure 5C). In the YUMM1.7 model, an initial study showed potent and dramatic activity for Cobi+Reg, such that complete responses were observed in 100% of treated mice that persisted (in the absence of ongoing therapy) without recurrence for greater than 30 days in 75% of the cases (Figure 5D and Supplemental Figure 5). Given these results, we aimed to determine whether Cobi+Reg could still be effective when treating more advanced tumors. As a result, we initiated Cobi+Reg therapy when the mean YUMM1.7 tumor volume exceeded 500 mm^3^ and observed complete responses in 87.5% of the mice (Figure 5E). We then assessed whether Cobi+Reg could produce tumor shrinkage after progression on ICB, in an attempt to mimic the clinical scenario whereby Cobi+Reg treatment would be administered after progression on ICB. B16F10 and YUMM1.7 tumor-bearing mice were treated with ICB until the tumors at least doubled in size, at which point ICB was discontinued and Cobi+Reg treatment was initiated. Cobi+Reg administration resulted in statistically significant tumor regression when compared with the vehicle control (Figure 5, F and G). In the multiple in vivo studies performed, the Cobi+Reg combination was well tolerated, without any overt signs of distress or weight loss in the treated mice. Analysis of serum chemistries identified mild elevations of aspartate aminotransferase in some of the treated mice (Supplemental Table 10), which is a known potential adverse event associated with regorafenib (31).

### Transcriptomic analysis of Cobi+Reg-treated tumors.

**Figure 6:**
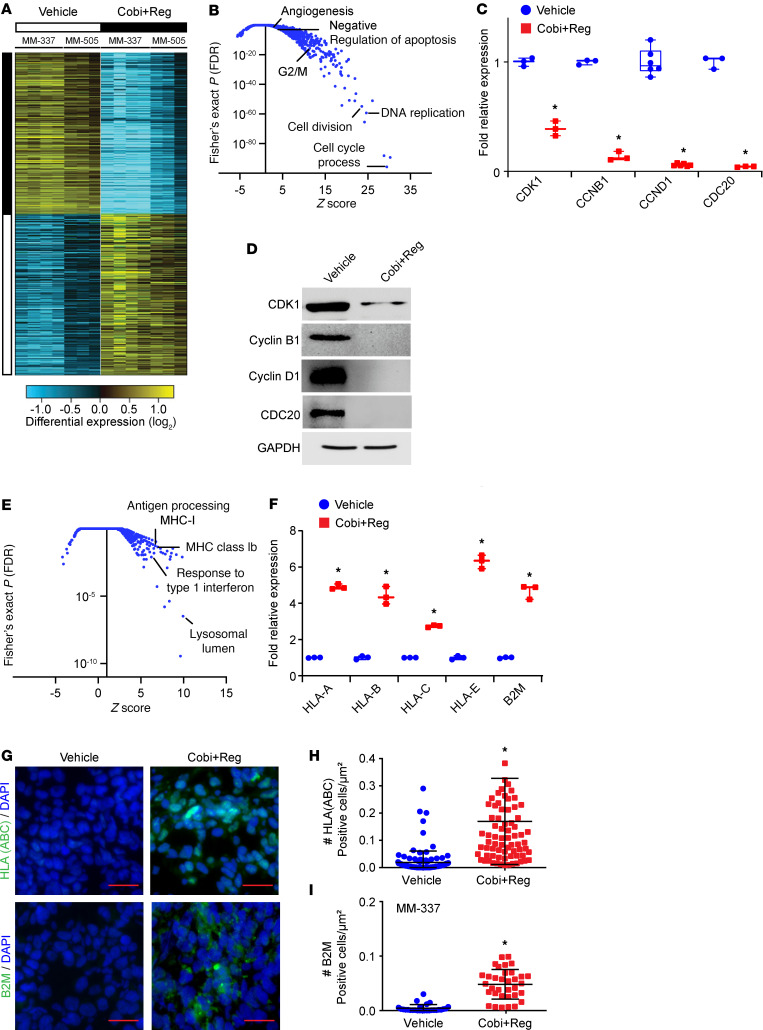
Identification of genes and cellular pathways regulated by cobimetinib plus regorafenib administration. (**A**) Heatmap of supervised analysis of RNA-Seq results of MM-337 and MM-505 in vivo tumors after treatment with vehicle or Cobi+Reg. (**B**) *z* scores of downregulated genes in various biological processes (as determined by Fisher’s exact test) identified by Gene Ontology analysis. (**C**) qRT-PCR analysis of expression of various differentially downregulated genes after treatment of MM-337 cells in culture with vehicle or Cobi+Reg; *P < 0.05 by Student’s *t* test. (**D**) Western blot analysis of expression of various proteins in MM-337 cells treated with vehicle or Cobi+Reg in culture. (**E**) *z* scores of upregulated genes in various biological processes identified by Gene Ontology analysis. (**F**) qRT-PCR analysis of expression of various differentially upregulated genes after treatment of MM-337 cells in culture with vehicle or Cobi+Reg; *P < 0.05 by Student’s *t* test. (**G**–**I**) Representative images of immunofluorescence detection, as well as quantification of expression of HLA (ABC) (**H**) and B2M (**I**) in MM-337 in vivo tumors treated with vehicle or Cobi+Reg; *P < 0.05 by Student’s *t* test. Scale bar: 20 μm.

Based on the substantial antitumor activity, including tumor regression, produced by Cobi+Reg treatment across multiple preclinical models, we sought to better understand its mechanism of action. We performed bulk RNA-Seq of Cobi+Reg-treated tumors in the MM-337 and MM-505 models. Supervised hierarchical analysis identified 614 statistically significantly differentially upregulated and 868 downregulated genes (Figure 6A and Supplemental Table 11). Gene Ontology analysis identified the following statistically significantly downregulated pathways, including several initially identified in ICB-resistant tumors: cell cycle (including M phase), cell division, DNA replication, angiogenesis, and negative regulation of apoptosis (Figure 6B). Among the downregulated genes were several involved in cell cycle progression, including *CCNB1*, *CCND1*, *CDK1*, and *CDC20*. The downregulation of these genes was confirmed at the RNA level by qRT-PCR analysis (Figure 6C and Supplemental Figure 6, A and B) and at the protein level by Western blot analysis (Figure 6D and Supplemental Figure 6C).

Surprisingly, Gene Ontology analysis identified upregulation of pathways involving antigen processing, MHC class Ib, and response to type 1 IFN (Figure 6E), given the differential overexpression of several MHC family gene members (*HLA*-*B*, *HLA*-*C*, and *HLA*-*E*). In addition, we assessed expression levels of *HLA-A* and *B2M*, a component of the class I MHC complex that plays an important role in antigen presentation and is reportedly lost after resistance to ICB (32). The statistically significant overexpression of these immunoregulatory genes was confirmed at the RNA level both in vivo and in culture (Figure 6F and Supplemental Figure 6D and E). Immunofluorescence analysis confirmed this upregulation, as the immunopositivity for HLA (ABC) and B2M was increased both in vivo and in culture after treatment with Cobi+Reg (Figure 6, G and H, and Supplemental Figure 7).

**Figure 7:**
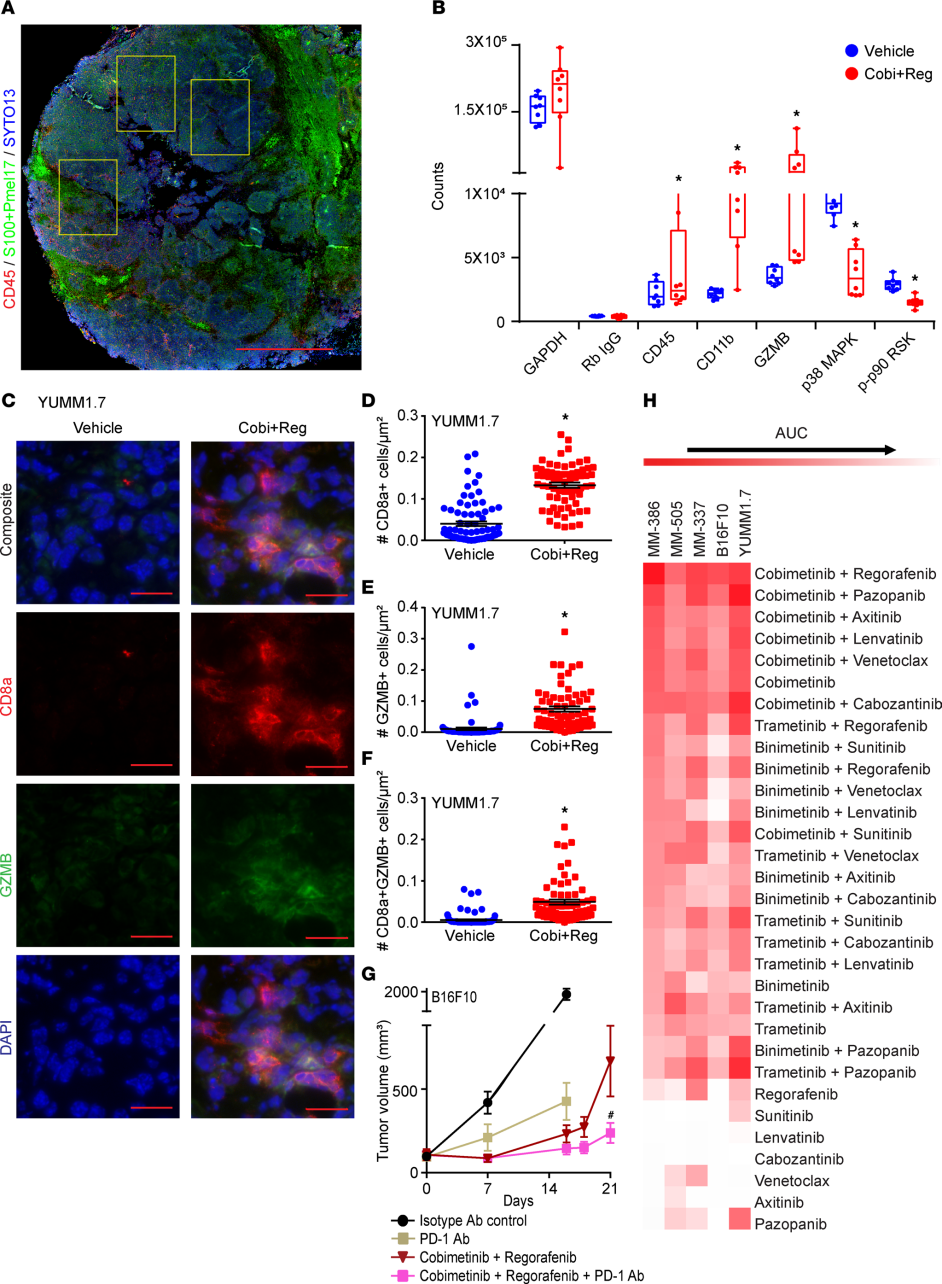
Upregulation of antigen presentation gene signature and T cell activation after cobimetinib plus regorafenib treatment and identification of additional active MEK inhibitor–VEGF inhibitor combinations. (**A**) Representative region of interest (yellow rectangle, up to 700 μm^2^) for multiplex digital spatial profiling analysis composed of B16F10 tumor cells (S100+Pmel17 stain, green) and a peritumoral zone to include immune cells (CD45+, red) along with SYTO13 (DNA, blue). Scale bar: 1 mm. (**B**) Results of multiplex digital spatial profiling analysis showing differential expression of various immune and tumor cell markers after treatment with vehicle or Cobi+Reg; *P < 0.05 by Student’s *t* test. (**C**) Representative images of immunofluorescence detection of expression, as well as quantification of expression of CD8a (**D**), granzyme B (**E**), or both proteins (**F**) in YUMM1.7 in vivo tumors treated with vehicle or Cobi+Reg; *P < 0.05 by Student’s *t* test. Scale bar: 20 μm. (**G**) Antitumor activity of various drugs on B16F10 melanoma in vivo; ^#^*P* < 0.05 by randomization test for comparison of Cobi+Reg versus Cobi+Reg + PD-1 Ab. (**H**) Heatmap of high-throughput drug screening analysis showing effects on cell viability of various single drugs and drug combinations in MM-386, MM-505, MM-337, B16F10, and YUMM1.7 cells.

In addition, we performed multiplex digital spatial profiling analysis of B16F10 in in vivo tumors treated with Cobi+Reg to examine the extent to which combinatorial drug therapy modified the tumor microenvironment (Figure 7A). Treatment of immunocompetent mice bearing B16F10 tumors led to upregulation of immune marker cells within the tumor microenvironment, along with downregulation of markers associated with MAPK pathway signaling. Specifically, this analysis detected increased expression of CD45 (reflecting the total immune population), as well as CD11B and granzyme B (GZMB) (representing markers of activated T cells), while revealing decreased expression of the MAPK markers p38 and phosphor-p90RSK (Figure 7B).

Taken together, these results suggest that Cobi+Reg treatment can improve antigen presentation, thereby activating a repertoire of immune cells that can mediate an antitumor response. To investigate this further, we assessed various T cell subsets in B16F10 and YUMM1.7 melanoma tumors in vivo after treatment with Cobi+Reg. Immunofluorescence analysis indicated statistically significant increases in the total CD8a-positive population in Cobi+Reg-treated tumors (Figure 7, C and D, and Supplemental Figure 8A). In addition, there was a statistically significant increase in the activated T cell population (as evidenced by levels of granzyme B–positive cells) (Figure 7, C and E, and Supplemental Figure 8, A and C). Accordingly, there was a marked increase in the CD8a and granzyme B “double-positive” T cell subset (Figure 7, C and F, and Supplemental Figure 8, A and D). Thus, the upregulation of the HLA gene family as well as *B2M* after Cobi+Reg treatment promoted a functional redistribution of the intratumoral T cell population, resulting in a shift toward activated T cell subsets. As a result of this finding, we hypothesized that treatment with Cobi+Reg in combination with PD-1 blockade may lead to enhancement of antitumor efficacy compared with the individual treatments. We tested this hypothesis in the B16F10 model and observed statistically significantly improved antitumor activity with the triple combination when compared with Cobi+Reg treatment (Figure 7G).

### Identification of additional active MAPK inhibitor–VEGF inhibitor combinations.

Finally, we explored whether combined targeting of MAPK and angiogenic pathways could more broadly recapitulate the antitumor activity observed. We developed an additional HTDS platform consisting of clinically approved MEK inhibitors and several multi-kinase inhibitors with antiangiogenic (including anti-VEGF) properties. Testing of all available combinations in the 3 PDX lines and 2 murine lines in culture showed a range of activity for the various combinations tested (Figure 7H and Supplemental Table 12). Intriguingly, the cobimetinib-containing combinations proved the most active when compared with other MEK inhibitor pairings. While Cobi+Reg consistently ranked among the most effective treatments, cobimetinib plus pazopanib (termed Cobi+Paz) emerged as another promising combinatorial treatment. The in vivo activity of Cobi+Paz was demonstrated in the YUMM1.7 model (Figure 5D and Supplemental Figure 5), including after progression on ICB (Figure 5G), and was comparable to that produced by Cobi+Reg. Thus, both Cobi+Reg and Cobi+Paz treatment were highly active in immunotherapy-insensitive murine models, including after progression on ICB.

## Discussion

In this study, we aimed to develop a combinatorial therapeutic approach to target ICB-resistant melanoma. RNA-Seq analysis of ICB-resistant metastatic melanoma tumors identified multiple potentially druggable genes and pathways. An HTDS targeting these pathways identified several active drug combinations that were validated in vivo in multiple PDX models encompassing the major molecular subtypes of melanoma derived from patients who progressed on PD-1–based ICB therapy. The Cobi+Reg combination emerged as the lead candidate and was further validated in immunocompetent murine melanoma models, including after progression on ICB therapy. RNA-Seq and spatial analysis of Cobi+Reg-treated tumors indicated upregulation of genes that promote antigen presentation and the adaptive immune response, which was accompanied by increased intratumoral activated T cell subsets, helping to promote increased activity of triple drug therapy (Cobi+Reg with PD-1 blockade) in the B16F10 model.

Our results are noteworthy for several reasons. To begin with, our study utilized the biology of ICB-resistant melanoma to identify therapeutic vulnerabilities. Bulk RNA-Seq analysis identified several differentially expressed genes involved in key protumorigenic pathways, including angiogenesis, MAPK signaling, antiapoptosis, and glycolysis that could explain the persistent survival of melanoma cells after treatment with ICB. Our results serve to extend the information provided by multiple prior studies that have defined the molecular landscape of melanoma in the setting of ICB resistance. These studies have demonstrated the contribution of various genetic programs or signaling pathways to immunotherapy resistance, including activation of angiogenesis (33) and cell cycle (specifically CDK4/6) (34), along with inactivation or loss of PTEN (35), β-catenin (36), and melanocytic antigen expression (associated with an undifferentiated signature) (37, 38). Separately, it is well appreciated that defects in IFN receptor signaling as well as antigen processing and presentation are an important component of ICB resistance (32, 37, 38), including mutations in HLA genes (e.g., *HLA-A/B/C)* and *B2M* (32, 34, 38). Intriguingly, Cobi+Reg treatment resulted in reversal of several of these resistance mechanisms, including suppression of angiogenesis and cell cycle progression as well as activation of MHC class I complex genes and *B2M*, providing a mechanistic basis for the antitumor activity produced, along with the immune activation promoted by this targeted combinatorial regimen.

The transcriptomic profiles of ICB-resistant melanoma formed the basis for designing an HTDS platform to identify targeted agents with potential antitumor activity. The drugs selected included those that impinged on the pathways identified by RNA-Seq analysis as well as drugs that targeted known pathway vulnerabilities present in melanoma cells. Several active combinations were identified by this analysis both in culture and in vivo, indicating the robustness of the HTDS platform. Specifically, Cobi+Reg and Cobi+Ven were shown to have substantial antitumor activity in multiple PDX models of ICB-resistant melanoma and were shown to be more active than either of the agents when administered alone. Overall, the Cobi+Reg combination emerged as a particularly promising combination, with marked in vivo antitumor activity demonstrated against ICB-resistant PDX models encompassing the major molecular melanoma subtypes (i.e., *BRAF*-, *NRAS*-, and *NF-1*–mutant) and after progression on combined ICB in immunocompetent murine melanoma models. Of note were the complete tumor regressions observed in the YUMM1.7 model, a more clinically relevant murine model that harbors important molecular aberrations observed in human melanoma (including in *Braf V600E*, and inactivated *Cdkn2a* and *Pten*) (39). Cobi+Reg administration resulted in a high proportion of durable complete responses, even when combinatorial therapy was initiated at a highly advanced tumor volume (>500 mm^3^). Importantly, Cobi+Reg therapy resulted in marked suppression of MAPK pathway signaling, along with reduced secretion of VEGF, which were concomitantly associated with altered proliferative, apoptotic, and angiogenic indices in treated tumor cells. Thus, a therapeutic approach that was effective in treating ICB-resistant melanoma successfully reversed key hallmarks of the biology of resistant tumors identified in patient specimens.

Taken together, our results identify Cobi+Reg as a promising therapeutic combination for salvage of advanced melanoma after progression on ICB. In addition, these results show that triple therapy (that included PD-1 blockade) resulted in increased antitumor efficacy, suggesting the potential utility of this regimen in the therapy of treatment-naive advanced melanoma. To our knowledge, this combinatorial approach has not been investigated clinically in any malignancy. Beyond Cobi+Reg, our expanded HTDS screen identified several intriguing combinations, including MEK inhibitors and angiogenesis inhibitors, reinforcing the importance of these pathways to the survival of ICB-resistant melanoma. This was supported by the substantial activity of Cobi+Paz in vivo, including after progression on ICB. Overall, given the activity of several combinations in the setting of ICB resistance, our studies provide a rational framework for clinical trial design focused on this treatment-refractory patient population, including a compelling rationale for a prospective clinical trial examining the activity of one or more of the regimens identified herein (e.g., Cobi+Reg, Cobi+Paz, or Cobi+Ven) after progression on PD-1–based immunotherapy, or of Cobi+Reg with PD-1 blockade in the advanced metastatic melanoma setting.

An unanticipated finding of this study was the observation of immune activation after Cobi+Reg treatment in vivo suggested by both RNA-Seq and spatial analyses of treated tumors. However, this observation is supported by prior studies demonstrating that MEK inhibition alone can exert proimmunogenic effects (40, 41), including the intratumoral recruitment of CD8^+^ T cells, resulting in potentiating responses to PD-1 blockade. Separately, regorafenib has also been shown to potentiate responses to PD-1 blockade, in part by promoting a CD8^+^ T cell infiltrate (42). However, our studies uniquely indicate the importance of the Cobi+Reg-mediated transcriptomic profile to activate expression of MHC class I genes and *B2M* in the promotion of an activated T cell response, resulting in sensitizing to ICB therapy.

In conclusion, our studies exploit the biology of melanoma resistant to ICB to identify several active drug combinations, specifically introducing Cobi+Reg after progression on immunotherapy or combined with ICB in the treatment-naive setting.

## Methods

### Sex as a biological variable

In the RNA-Seq analysis of patient samples, our study examined specimens from both males and females. Our animal studies exclusively examined female mice. It is unknown whether the findings are relevant for male mice.

### PDX model development and cell culture conditions

In-house PDX generation and cell culture conditions were previously described (27, 43). Briefly, PDXCs (MM-337, MM-386, MM-505, MM-507, MM-567, MM-574, MM-578, MM-363, MM-313, MM-348, MM-425, and MM-309) were cultured as neurospheres in ultralow attachment T25 flasks with DMEM/Ham’s F12 medium containing 1× B27 supplement (Gibco), 1× penicillin-streptomycin, 50 ng/mL EGF, 50 ng/mL FGF, and no serum. Short tandem repeat analysis of PDX lines was performed by ATCC for authentication. All PDX lines were reported to be human, with no matches in the ATCC database.

### Murine melanoma model cell culture

Murine melanoma cell lines B16F10 (purchased from ATCC) and YUMM1.7 (provided in-house) were grown in DMEM F12 with 5% FBS (Invitrogen Life Technologies). Cell culture media was supplemented with 1× penicillin/streptomycin (Thermo Fisher Scientific) and1× of MEM Non-Essential Amino Acids Solution (100×) (Gibco), and cells were grown at 37°C and 5% CO_2_. All cell lines were routinely tested for mycoplasma contamination using MycoFluor Mycoplasma Detection kit (Thermo Fisher Scientific) following the manufacturer’s instructions.

### Pharmacological studies and HTDS

#### Development of inhibitor screen.

All PDXCs were plated in 384-well round-bottom microplates and allowed to acclimate for 3 days for formation of tumor-spheres before addition of drugs. Individual drugs and combinations were dispensed using Beckman Coulter’s Echo Liquid Handler and cell viability was read 72 hours later. PDXCs were screened as previously described (27) in an HTDS format against a 6-point concentration-response curve of drugs chosen primarily based on the following criteria: (a) The drug is FDA-approved or in clinical trials; (b) The drug is available for research purposes; (c) Pharmacokinetic data in humans are available. For most drugs, the 6-point concentration-response curve contained drug concentrations beginning with the highest concentration starting with the approximate C_max_ reported in published clinical trials (Supplemental Table 2). As part of our drug evaluation platform, all the analyses were automated using specialized in-house Visual Basic for Applications–programmed Excel spreadsheets and GraphPad Prism. All drugs were obtained through Selleck Chemicals.

#### Quantification of drug response.

The percentage of cell viability was equal to Treatment_A_/Control_A_ × 100%, where A equals absorbance. The resulting drug-response data were used to calculate AUC and corresponding confidence limits with GraphPad Prism using the formula ΔX*(Y1 + Y2)/2 as previously described (27). Drug combinations were further evaluated using the program CompuSyn (www.combosyn.com; PD Science, LLC). A combination index value of less than 1 indicates synergism, equal to 1 is additive effect, and greater than 1 is antagonism (28).

### RNA extraction and qRT-PCR

Cells were treated with DMSO or Cobi (0.5 μM) + Reg (5 μM) for 48 hours. RNA extraction, cDNA synthesis, and qRT-PCR were performed as described previously (44). TaqMan probes for the various genes assayed were purchased from Thermo Fisher Scientific: *HLA-A* (Hs01058806_g1; 4331182), *HLA-B* (Hs07292706_g1; 4351372), *HLA-C* (Hs00740298_g1; 4331182), *HLA-E* (Hs03045171_m1; 4331182), *B2M* (Hs00187842_m1; 4331182), *CDC20* (Hs00961702_g1; 4351372), *CDK1* (Hs00938777_m1; 4331182), *CCND1* (Hs00765553_m1; 4331182), *CCNB1* (Hs01030099_m1, 4331182), *CD44* (Hs01075864_m1; 4331182), *HK2* (Hs00606086_m1; 4331182), *HPSE* (Hs00180737_m1; 4331182), *NRAS* (Hs00180035_m1; 4331182), *MCL1* (Hs01050896_m1; 4331182), *FN1* (Hs0036505052_m1; 4331182), *GAPDH* (Hs02786624_g1; 4331182), and *HPRT1* (Hs02800695_m1; 4331182).

### RNA-Seq and bioinformatics data analysis

RNA extraction from flash-frozen human or murine tissue samples was performed as previously described (45–47). RNA-Seq was performed from approximately 500 ng of total RNA processed using TruSeq polyA selection, at a target depth of 40 million paired-end, stranded reads on an Illumina 2500. 

### Gene expression analyses

The RNA-Seq data was analyzed as previously described (43, 48), initially aligned to the human reference genome (hg19) using the software STAR, followed by gene quantification in the software AltAnalyze to obtain gene-level RPKM values. Gene expression quantification (RPKM) was determined from exon-exon junctions and differential expression (fold ≥1.5, empirical Bayes moderated *t* test *P* < 0.05) was performed in AltAnalyze version 2.1.3 using the Ensembl 72 human database. Embedded gene-set enrichment analyses were performed using GO-Elite with default options. Hierarchical clustering was performed in AltAnalyze using HOPACH clustering for rows and weighted cosine clustering for genes.

### Caspase 3/7 assay

Cells were incubated with DMSO, Cobi (0.5 μM), Reg (5 μM), or Cobi (0.5 μM) + Reg (5 μM) for 48 hours. The caspase 3/7 assay was performed by using the Muse Caspase-3/7 kit (EMD Millipore) following the manufacturer’s instructions.

### Western blot analysis

Western blot analysis was performed as described previously (49, 50). Cells were treated with DMSO or Cobi (0.5 μM) + Reg (5 μM) for 48 hours. Next, 50 μg of protein was electrophoresed in 10% Tris-HCl denaturing gels (Bio-Rad). Target proteins were detected by using specific antibodies against ERK1/2 (catalog 4695), pERK1/2 (catalog 9106), BCL2 (catalog 4223), tMEK (catalog 8727), pMEK (catalog 9154), tRSK (catalog 8408), pRSK (catalog 11989), and CDC20 (catalog 4823) from Cell Signaling Technology. The CDK1 (A303-663A) antibody was purchased from Thermo Fisher Scientific. Antibodies targeting cyclin D1 (sc-8396), cyclin B1 (sc-7393), and GAPDH (sc-365062, used as a loading control) were purchased from Santa Cruz Biotechnology.

### IHC

IHC analysis was performed as previously described (44, 51). The CONFIRM anti–Ki-67 30-9 antibody (790-4286, prediluted, Ventana Medical Systems) was used for staining by employing the Ventana Benchmark autostainer (Ventana Medical Systems). Ki-67 staining is reported as the average staining intensity from 7 randomly selected tumor-containing regions. IHC analysis of CD31 immunostaining was performed using the rabbit CD31 antibody (ab56299 at 1:50 dilution, Abcam) as previously described (52), followed by 1-step polymer-HRP IHC detection system (Biogenex). The entire tumor-containing area was analyzed at 40× magnification with the Mirax Midi Digital Platform (Zeiss). The number of CD31-positive vessels was manually counted and the number of lumen-containing vessels reported.

### Immunofluorescence analysis

Quantification of protein expression using immunofluorescence was performed on cells cultured on coverslips and FFPE tissue sections as previously described (53, 54). FITC-conjugated antibodies against HLA (ABC) (catalog 311403) and B2M (catalog 395705) from BioLegend, each at 1:500 dilution, were used to test the upregulation of protein expression. Alexa Fluor 594 conjugated CD8a (catalog 126405) and FITC-conjugated granzyme B (catalog 515403) antibodies were used at 1:500 dilution to detect the signature of T cell activation proteins. Images were taken at fixed exposures with a Zeiss Axio Imager Z2 microscope, and the fluorescence intensities of individual cells were quantified using Zeiss AxioVision. The mean pixel intensities were used for statistical analysis using Microsoft Excel and GraphPad Prism. The expression data were quantified as the amount of fluorescence per single nucleus.

### Antibodies

The antibodies used in this study were as follows: FITC anti-human β2-microglobulin antibody (BioLegend 395706, RRID:AB_2801055), FITC anti-human HLA-A, B, C antibody (BioLegend 311404, RRID:AB_314873), Alexa Fluor 594 anti-mouse CD8a antibody (BioLegend 100758, RRID:AB_2563237), FITC anti-human/mouse granzyme B antibody (BioLegend 515403, RRID:AB_2114575), p44/42 MAPK (Erk1/2) (137F5) rabbit mAb (Cell Signaling Technology 4695, RRID:AB_390779), phospho-p44/42 MAPK (Erk1/2) (Thr202/Tyr204) (E10) mouse mAb (Cell Signaling Technology 9106, RRID:AB_331768), Bcl-2 (D55G8) rabbit mAb (Cell Signaling Technology 4223, RRID:AB_1903909), MEK1/2 (D1A5) rabbit mAb (Cell Signaling Technology 8727, RRID:AB_10829473), phospho-MEK1/2 (Ser217/221) (41G9) rabbit mAb (Cell Signaling Technology 9154, RRID:AB_2138017), RSK1 (D6D5) rabbit mAb (Cell Signaling Technology 8408, RRID:AB_10828594), phospho-p90RSK (Ser380) (D3H11) rabbit mAb (Cell Signaling Technology 11989, RRID:AB_2687613), CDC20 antibody (Cell Signaling Technology 4823, RRID:AB_10549074), cyclin D1 antibody (A-12) (Santa Cruz Biotechnology sc-8396, RRID:AB_627344), CCNB1/cyclin B1 antibody (D-11) (Santa Cruz Biotechnology sc-7393, RRID:AB_627336), GAPDH antibody (G-9) (Santa Cruz Biotechnology sc-365062, RRID:AB_10847862), rabbit anti-CDK1 antibody (Thermo Fisher Scientific A303-663A, RRID:AB_11205291), CONFIRM anti–Ki-67 (30-9) rabbit monoclonal primary antibody (Ventana Medical Systems 790-4286, RRID:AB_2631262), and anti-CD31 antibody (RM0032-1D12) (Abcam ab56299, RRID:AB_940884).

### Multiplex spatial profiling analysis

Multiplex digital spatial profiling analysis was performed at the core facility at the Knight Cancer Institute, a Center of Excellence for the Nanostring GeoMx platform, after a mixture of antibodies for 17 immune and 10 tumor cell markers (https://nanostring.com/products/geomx-digital-spatial-profiler/geomx-dsp-overview/">https://nanostring.com/products/geomx-digital-spatial-profiler/geomx-dsp-overview/) tagged by a unique oligonucleotide via a UV-sensitive chemical linker was applied to the slide. Up to four regions of interest from 2 mouse tumors were selected from control and Cobi+Reg-treated mice and subjected to UV light pulsed on the cells of interest, releasing the oligonucleotides from the bound antibodies. The released oligonucleotides were auto-transferred to a 96-well plate and quantitated through hybridization and counting using the MAX nCounter system (nanoString, Bruker Spatial Biology). Within a region of interest (up to 700 μm^2^), collections were made from the tumor cells (S100 positive) and a peritumoral zone that included immune cells (CD45 positive). A tissue microarray of control samples (cancer cell lines and normal tissues) was analyzed during every run to ensure proper functioning of all antibodies and to allow normalization of samples across different runs and limit batch effects. Validation of antibodies included IHC or immunofluorescence staining comparisons between the original (untagged) and oligo-tagged versions, followed by performance testing of multiplexes of the tagged versions on control cell lines and tissues with known expression characteristics. Marker expression statistically significantly greater than the mean and standard deviation of the IgG controls were evaluated.

### ELISA

ELISA assays for VEGFA expression from the PDXC supernatants were performed by using the human VEGFA ELISA kits (RayBiotech, Inc.).

### Animal studies

Six- to 8-week-old NOD SCID gamma (NSG) (for testing of PDX models) and C57BL/6 (for testing of B16F10 and YUMM1.7 models) female mice were purchased from The Jackson Laboratory. Standard animal housing and husbandry was used. PDX (0.5 × 10^6^), B16F10 (1 × 10^5^), or YUMM1.7 (0.75 × 10^5^) cells were mixed with 50% Matrigel for s.c. injection in a total volume of 100 μL in the mouse flank. Once tumors were palpable, mice were randomized and divided into groups with average tumor volumes of 70 mm^3^ or greater. Mice were divided into the following treatment groups (n ≥ 6): vehicle or isotype antibody, cobimetinib, regorafenib, venetoclax, cobimetinib plus linsitinib, Cobi+Reg, cobimetinib plus vorinostat, Cobi+Ven, Cobi+Paz, anti-PD-1 antibody, anti–PD-1 plus anti–CTLA-4 antibody, and cobimetinib plus regorafenib plus anti-PD-1 antibody. The animals were randomly assigned to treatment groups, and the investigator performing tumor measurements was blinded to the identity of the treatment groups. No samples were excluded from the analysis. Toxicity studies were performed initially to determine the optimal tolerable dose for single agents and drug combinations. All drugs were administered i.p. at the following doses: isotype antibody (0.2 mg/mouse), PD-1 antibody (0.2 mg/mouse), CTLA-4 antibody (0.2 mg/mouse), cobimetinib (5 mg/kg), linsitinib (10 mg/kg), venetoclax (15 mg/kg), regorafenib (8 mg/kg), and pazopanib (5 mg/kg). All drugs were administered 5 times a week, whereas antibodies were administered 3 times a week. Tumors were measured by caliper, and volumes were calculated as a product of length × width^2^/2. Mice were euthanized and specimens collected and processed for further analyses. The assessment of potential toxicity of Cobi+Reg treatment was performed at the Comparative Pathology Lab at the UC Davis School of Veterinary Medicine.

### Statistics

All quantified data represent an average of at least triplicate samples or as indicated. Statistical significance was determined using the Student’s *t* test or randomization test with Bonferroni’s multiple-comparison test where applicable, 2-way ANOVA repeated measures and a Tukey’s multiple-comparison test, or Kolmogorov-Smirnov test. Two-tailed *P* values less than 0.05 were considered statistically significant. The IC_50_ values with corresponding 95% confidence limits were compared by the analysis of logged data using GraphPad Prism. To test for synergism, the combination index was calculated using CompuSyn where a combination index less than 1 indicates synergism, equal to 1 is additive effect, and greater than 1 is antagonism, as previously described (28, 55) and as previously published by our group (56).

### Study approval

Acquisition of samples from patients with melanoma was performed according to Declaration of Helsinki principles and under the auspices of a protocol approved by the Sutter Health IRB with informed consent from each patient. Animal studies were carried out in accordance with NIH guidelines, the Health Research Extension Act of 1985, and the Public Health Service Policy on Humane Care and Use of Laboratory Animals, Office of Laboratory Animal Welfare assurance, and an approved IACUC protocol.

### Data availability

All data from the present study are present in the Supplemental Tables, Supporting Data Values file, or from the corresponding author. RNA-Seq data that support the findings of this study have been deposited in the NCBI’s Gene Expression Omnibus database (GEO GSE264375).

## Author contributions

MKS and SM conceptualized the study, designed the experiments, analyzed the data, and prepared the initial draft of the manuscript. IK, ARB, VB, AD, DDS, RI, MN, and PYD performed experiments. JRM III performed statistical analysis of in vivo studies. AB and NS performed bioinformatic analysis of RNA-Seq results. MB, SPL, and KBK provided vital human specimens or mouse models for the study. LS, YS, JEC, and JMK provided key input into study design and data analysis. All authors contributed to reviewing and editing the manuscript. Authorship order was assigned by the senior authors of the manuscript after discussion with the co–first authors.

## Supplementary Material

Supplemental data

Unedited blot and gel images

Supplemental tables 1-12

Supporting data values

## Version 1 07/10/2025

In-Press Preview

## Version 2 09/16/2025

Electronic publication
