# Supplementary material for: Combinatorial therapy regimens targeting preclinical models of melanoma resistant to immune checkpoint blockade
Source: J Clin Invest. 2025 Jul 10;135(18):e185220. doi: 10.1172/JCI185220 (PMC12490269; doi:10.1172/JCI185220)
Supplement: Supplemental data [file jci-135-185220-s206.pdf]

Supplementary Figure 1

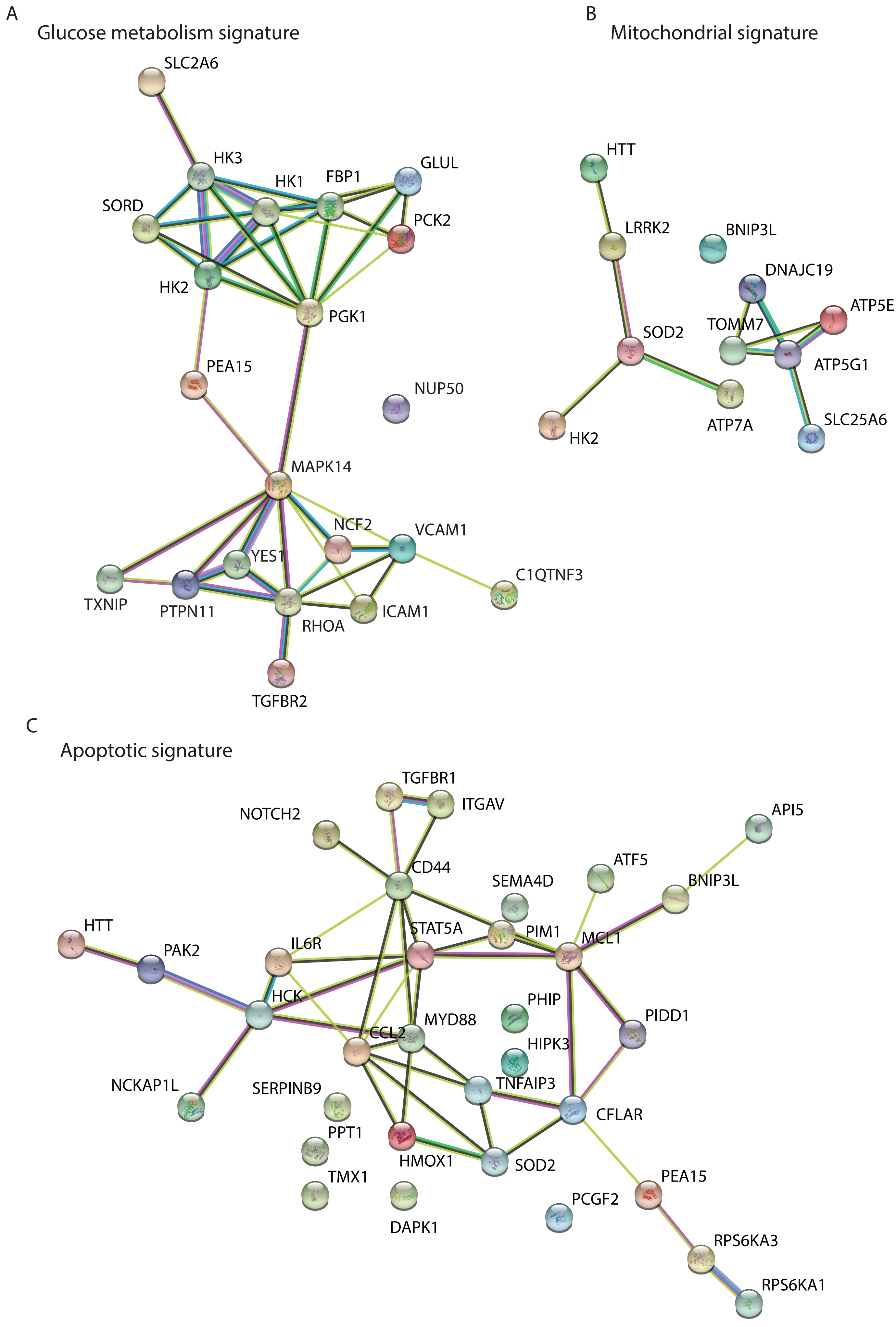

Supplementary Figure 2

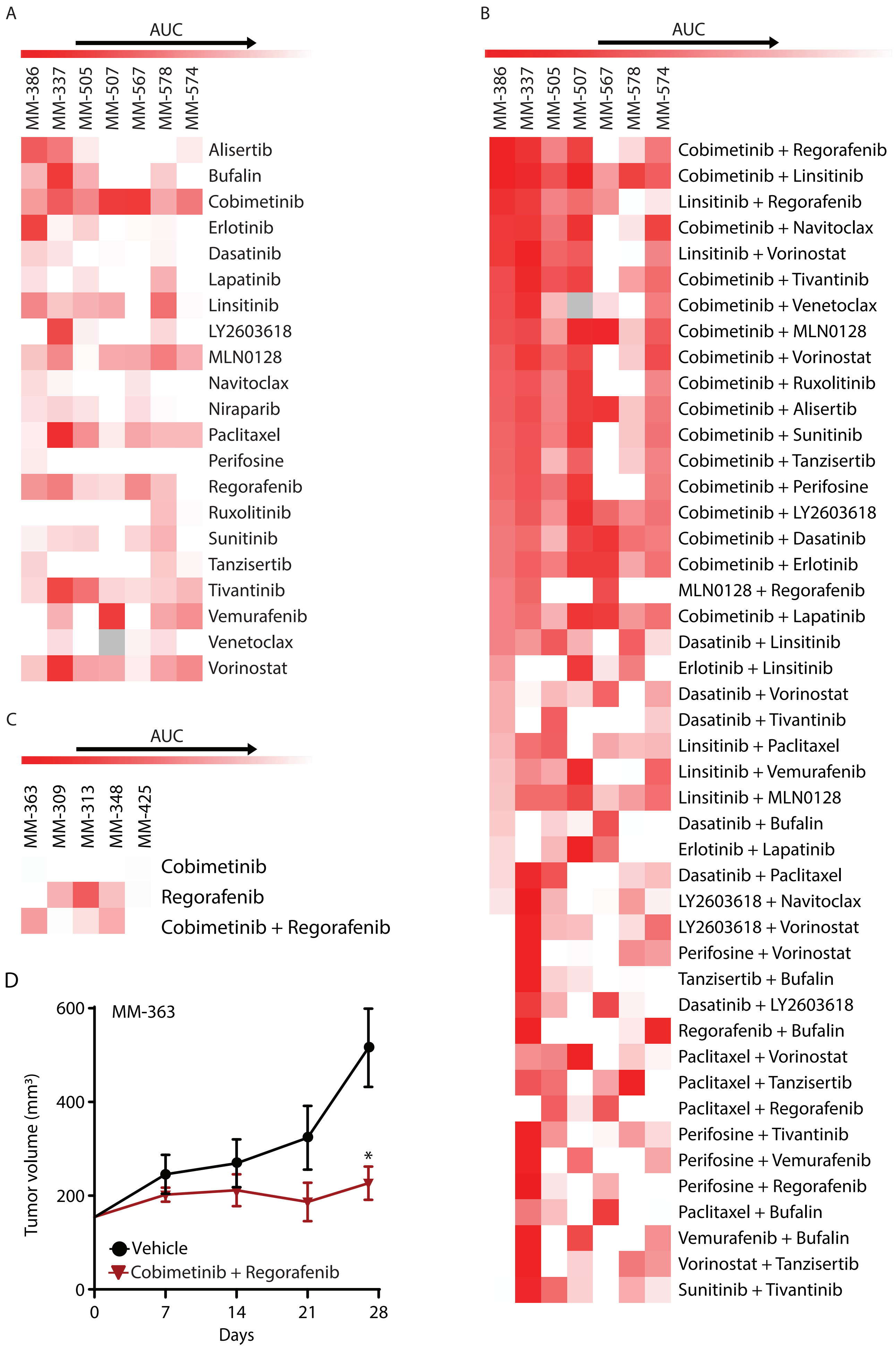

Supplementary Figure 3

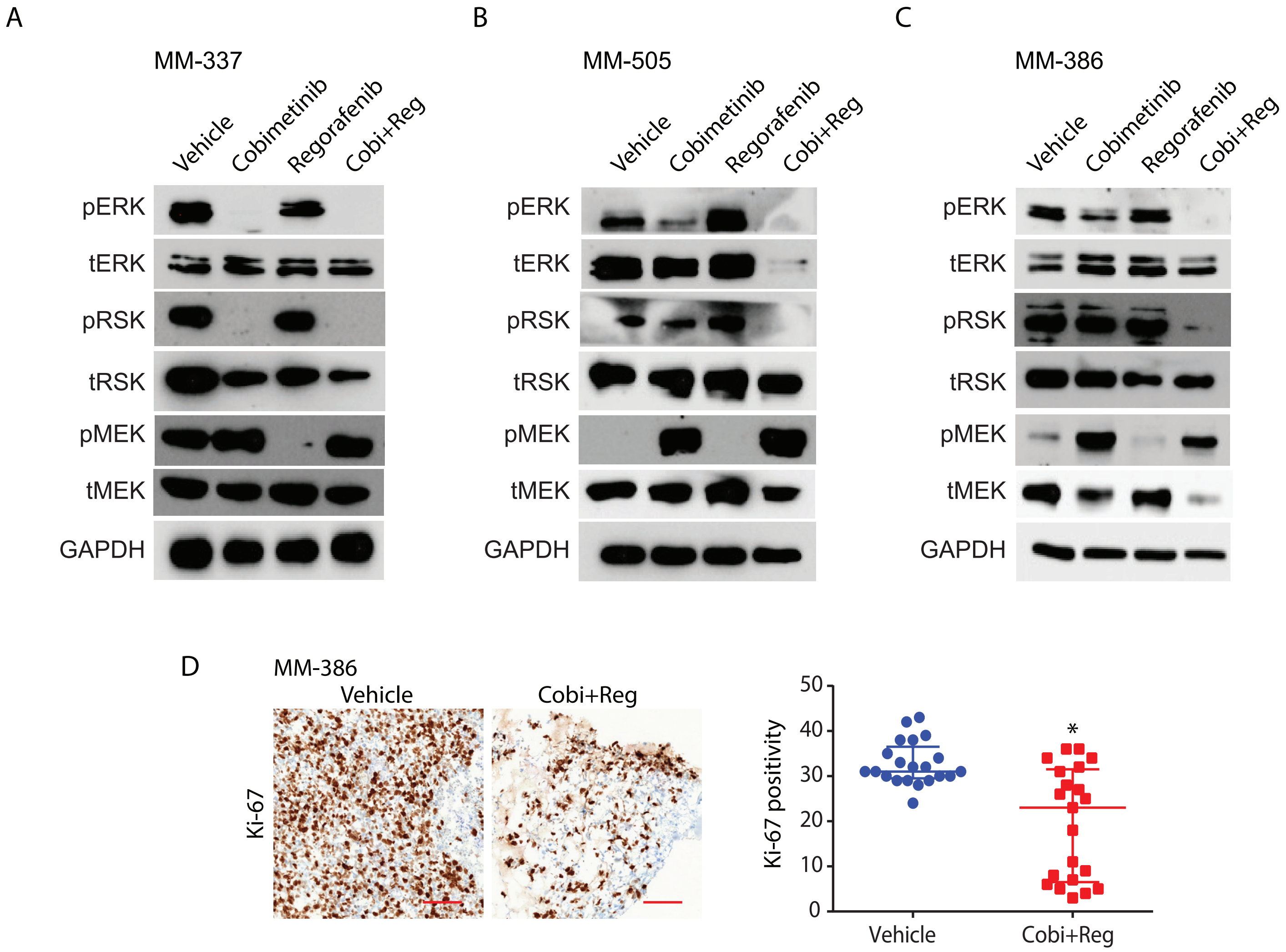

Supplementary Figure 4

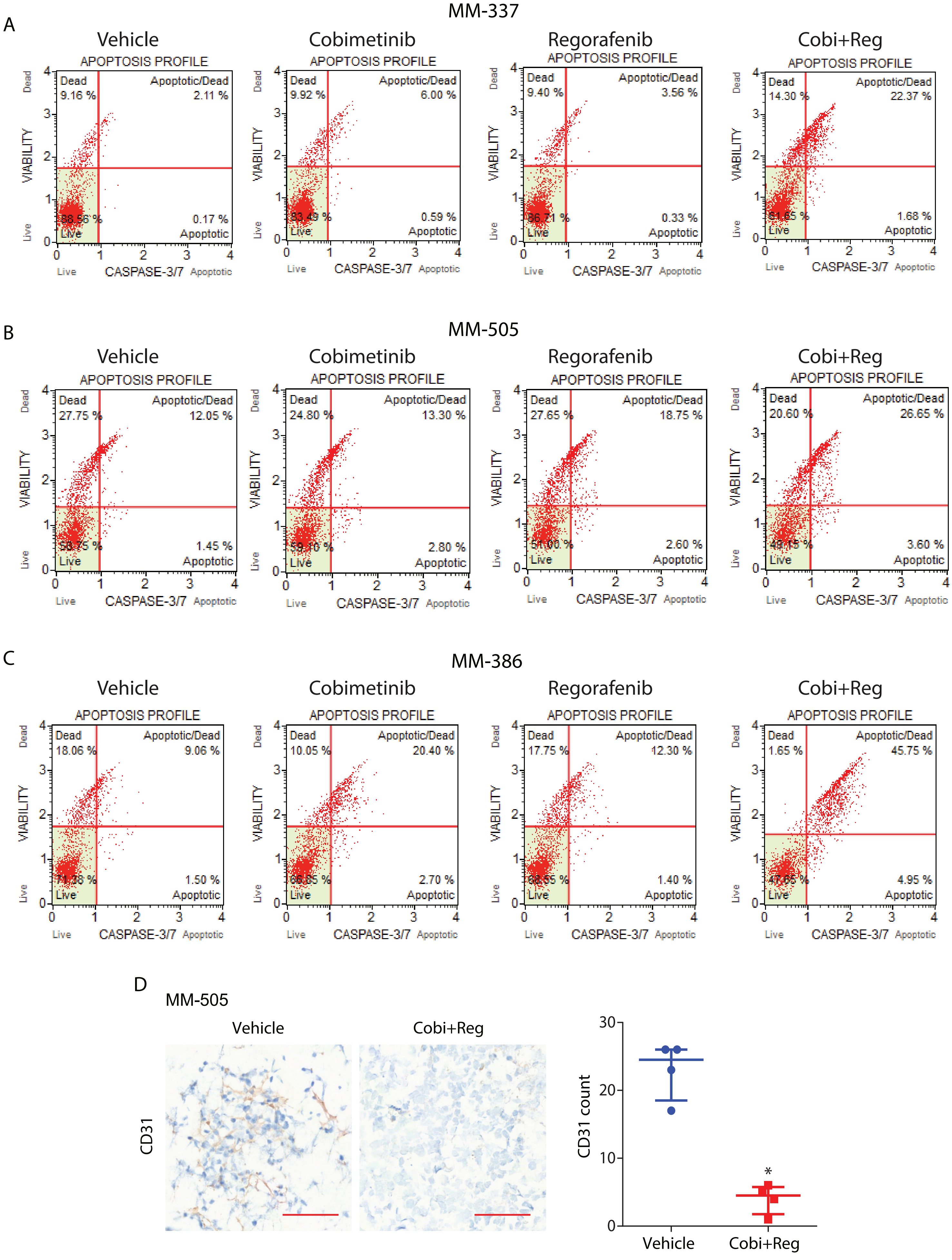

Supplementary Figure 5

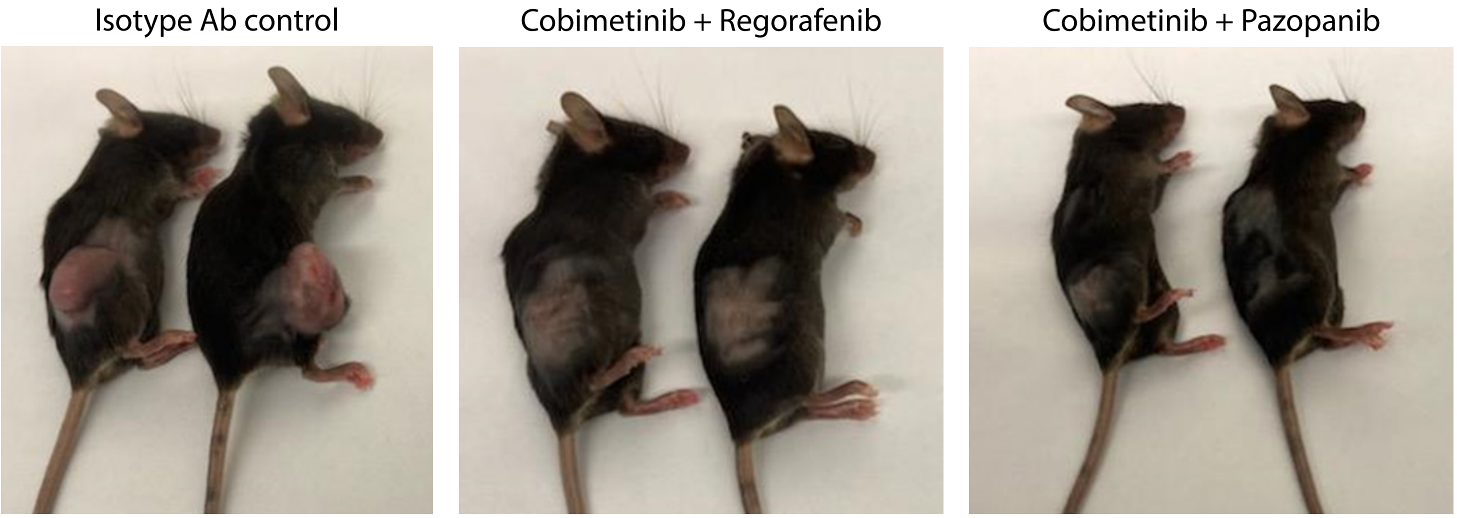

Supplementary Figure 6

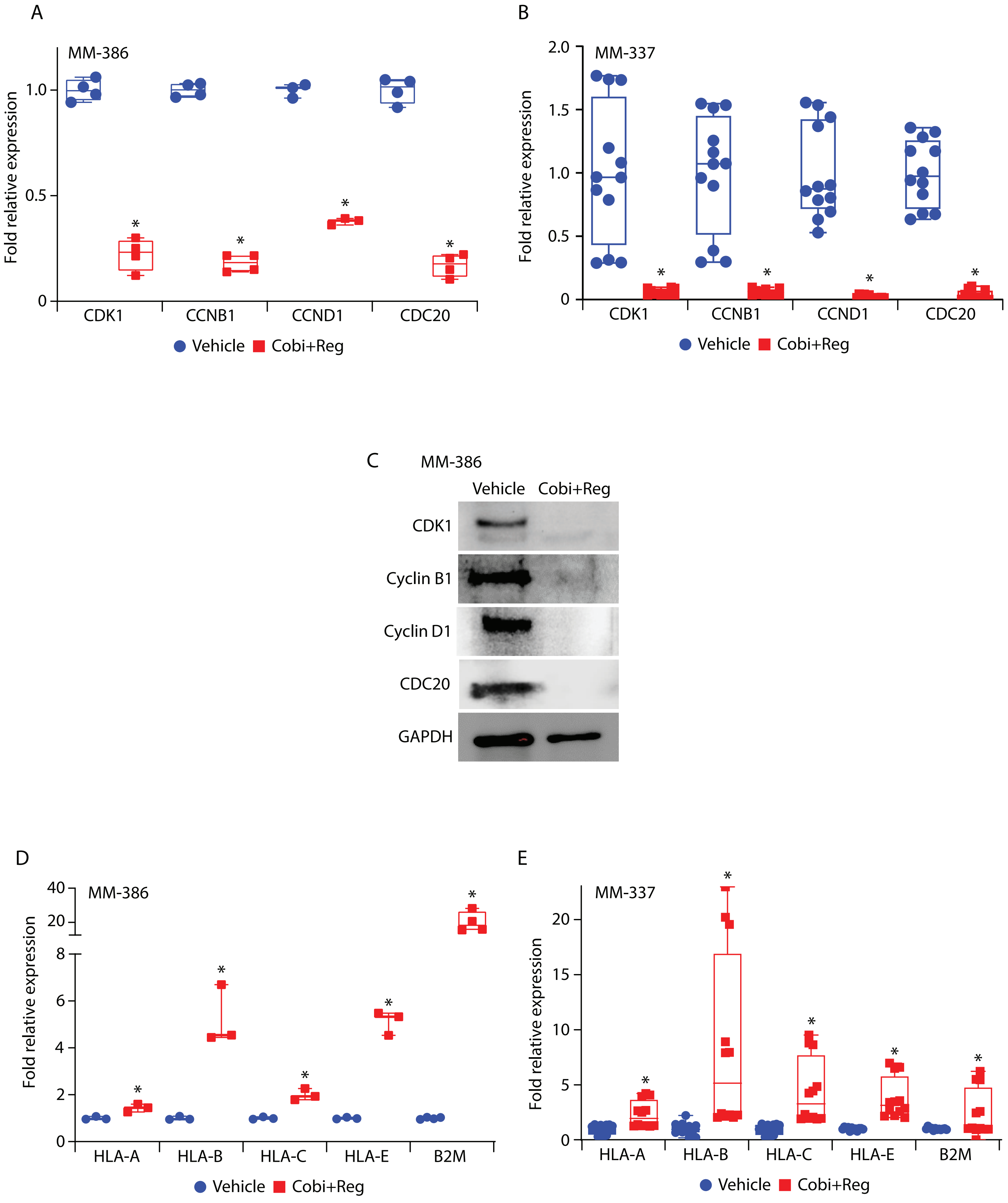

Supplementary Figure 7

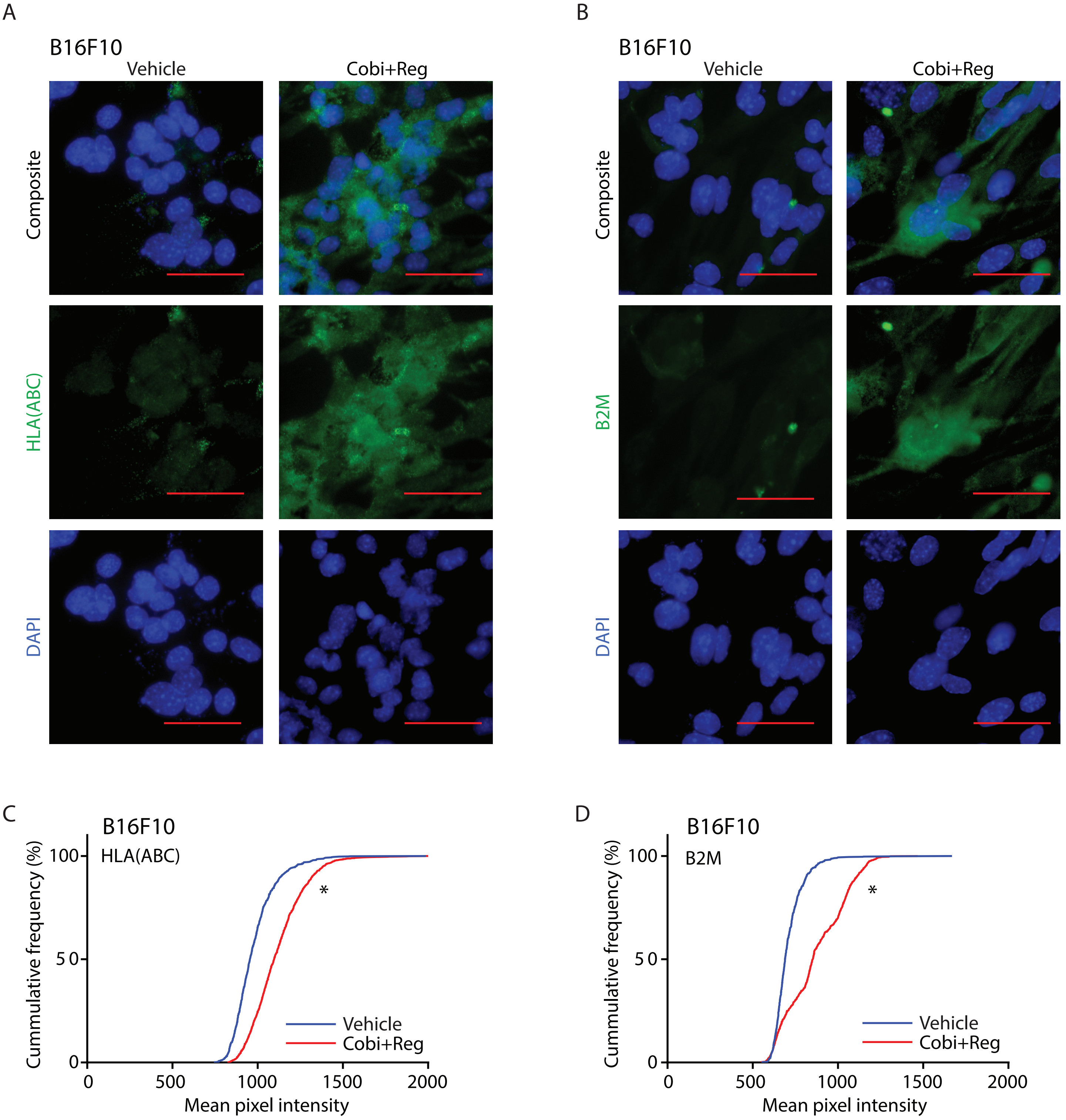

Supplementary Figure 8

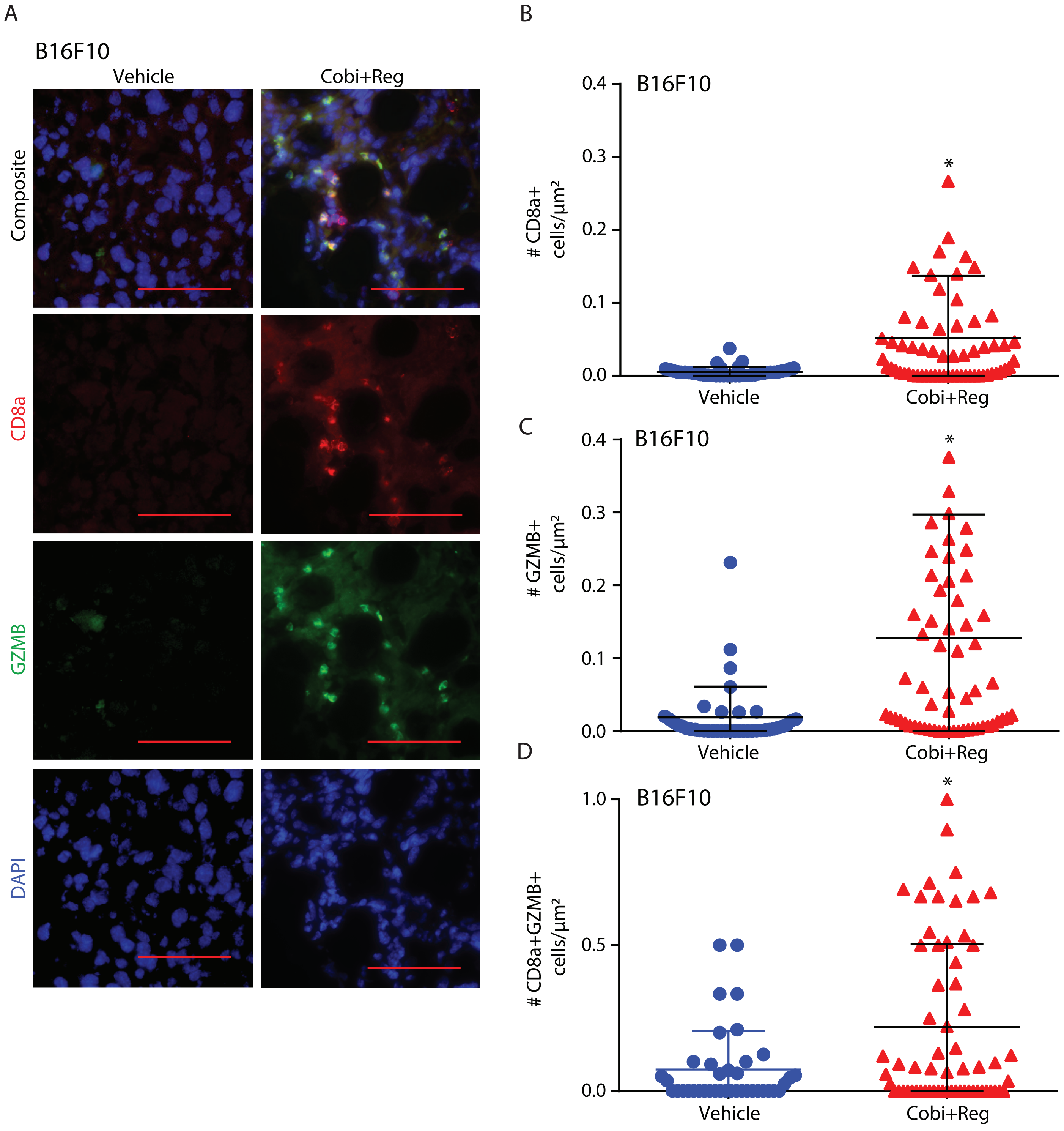

Supplementary Figure Legends

Figure S1. WikiPathways analysis of differentially expressed genes in ICB-resistant melanomas involved in the following biological processes: (A) glucose metabolism, (B) mitochondrial, and (C) negative regulation of apoptosis signatures.

Figure S2. Heatmap of HTDS analysis demonstrating the effects on cell viability of all single drugs alone (A) and in combination (B) in seven treatment-resistant PDXCs and a panel of five ICB-naïve human melanoma PDX lines (C). Increasing AUC (Area Under the treatment Curve) indicates decreasing sensitivity to the compound. (D) Tumor volumes of mice bearing MM-363 melanoma treated with vehicle or Cobi-Reg. *$P < 0.05$ by Student's t test for vehicle vs Cobi+reg.

Figure S3. Western analysis of expression of various MAPK pathway proteins following treatment of MM-337 (A), MM-505 (B), and MM-386 (C) with Cobi+Reg in culture. (D) Representative image and quantification of Ki-67 staining in MM-505 *in vivo* tumors treated with vehicle or Cobi+Reg. *$P < 0.05$ by Student's t test. Scale bar 50 µm.

Figure S4. Results of caspase 3/7 assay of apoptosis following treatment of MM-337 (A), MM-505 (B) and MM-386 (C) cells in culture with vehicle, Cobi, Reg, or Cobi+Reg. (D) Representative image and quantification of CD31 staining in MM-505 *in vivo* tumors treated with vehicle or Cobi+Reg. *$P < 0.05$ by Student's t test. Scale bar 50 µm.

Figure S5. Photographs of YUMM1.7 tumor-bearing C57BL/6 mice treated with vehicle, Cobi+Reg, or Cobi+Paz.

Figure S6. qRT-PCR analysis of various differentially downregulated genes following treatment with vehicle or Cobi+Reg in MM-386 *in vitro* (A) and MM-337 *in vivo* tumors (B). *$P < 0.05$ by 2-tailed unpaired Student's t test. (C) Western analysis of expression of various proteins in MM-386 cells in culture treated with vehicle or Cobi+Reg. (D-E) qRT-PCR analysis of various

differentially upregulated genes following treatment with vehicle or Cobi+Reg in MM-386 *in vitro* (D) and MM-337 *in vivo* tumors (E). *P < 0.05 by 2-tailed unpaired Student's t test.

Figure S7. Immunofluorescence analysis of expression of HLA(ABC) *in vitro.* Qualitative analysis of HLA(ABC) (A) and B2M (B) in B16F10 cells treated with vehicle or Cobi+Reg. Quantitative immunofluorescence analysis of expression of HLA(ABC) (C) and B2M (D) in B16F10 cells treated with vehicle or Cobi+Reg. *P < 0.05 by Kolmogorov-Smirnov test. Scale bar 20 μm.

Figure S8. Immunofluorescence analysis of expression of CD8a and GZMB *in vivo*. (A) Qualitative immunofluorescence analysis of expression of CD8a and GZMB in B16F10 *in vivo* tumors treated with vehicle or Cobi+Reg. Quantification of expression CD8a (B), GZMB (C) and double positivity of both proteins (D) in B16F10 *in vivo* tumors treated with vehicle or Cobi+Reg. *P < 0.05 by Student's t test. Scale bar 20 μm.
