# Supplementary material for: Combinatorial therapy regimens targeting preclinical models of melanoma resistant to immune checkpoint blockade
Source: J Clin Invest. 2025 Jul 10;135(18):e185220. doi: 10.1172/JCI185220 (PMC12490269; doi:10.1172/JCI185220)

Figure 1G

Phospho-p44/42 MAPK (Erk1/2) (Thr202/Tyr204) (E10) Mouse mAb (Cell Signaling Technology Cat# 9106, RRID:AB_331768)

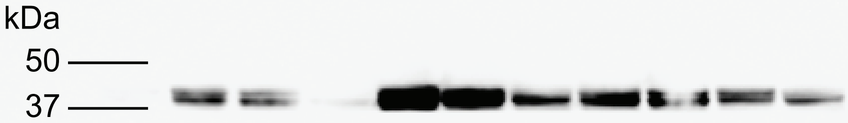

Figure 1G
Phospho-p44/42 MAPK (Erk1/2) (Thr202/Tyr204) (E10) Mouse mAb
(Cell Signaling Technology Cat# 9106, RRID:AB_331768)

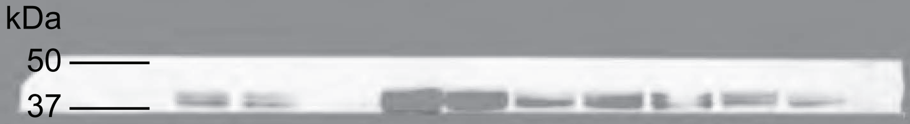

Figure 1G
p44/42 MAPK (Erk1/2) (137F5) Rabbit mAb
(Cell Signaling Technology Cat# 4695, RRID:AB_390779)

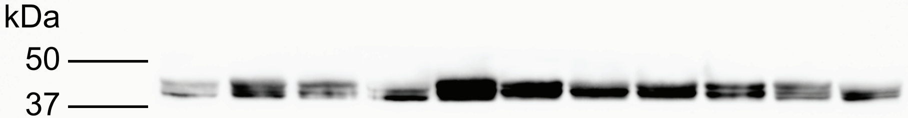

Figure 1G

Bcl-2 (D55G8) Rabbit mAb #4223

(Cell Signaling Technology Cat# 4223, RRID:AB_1903909)

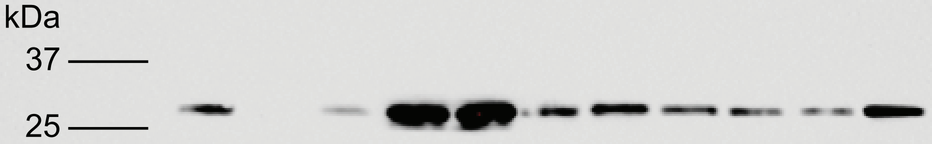

Figure 1G
GAPDH Antibody (G-9)
(Santa Cruz Biotechnology Cat# sc-365062, RRID:AB_10847862)

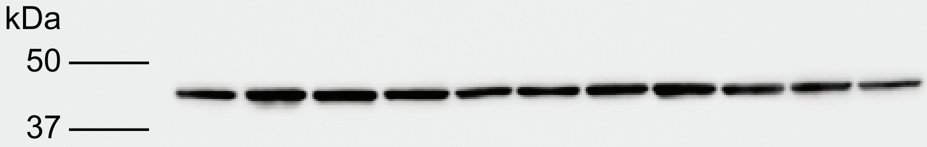

Figure 3D
Phospho-p44/42 MAPK (Erk1/2) (Thr202/Tyr204) (E10) Mouse mAb
(Cell Signaling Technology Cat# 9106, RRID:AB_331768)

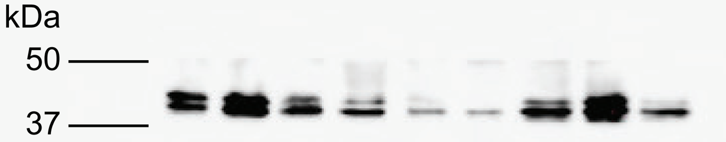

Figure 3D
p44/42 MAPK (Erk1/2) (137F5) Rabbit mAb
(Cell Signaling Technology Cat# 4695, RRID:AB_390779)

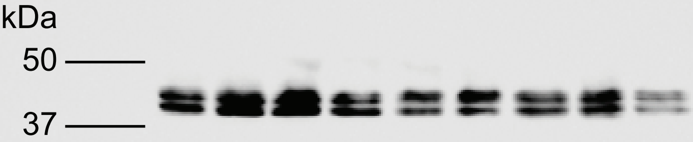

Figure 3D
Phospho-p90RSK (Ser380) (D3H11) Rabbit mAb
(Cell Signaling Technology Cat# 11989, RRID:AB_2687613)

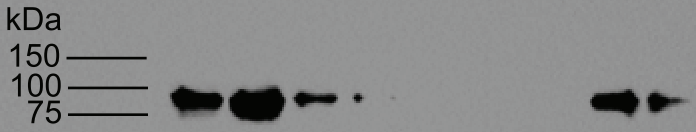

Figure 3D

Phospho-p90RSK (Ser380) (D3H11) Rabbit mAb

(Cell Signaling Technology Cat# 11989, RRID:AB_2687613)

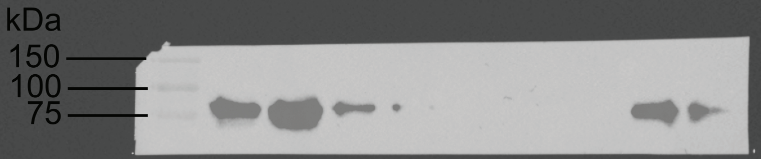

Figure 3D
RSK1 (D6D5) Rabbit mAb
(Cell Signaling Technology Cat# 8408, RRID:AB_10828594)

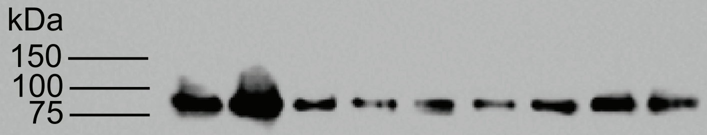

Figure 3D
Phospho-MEK1/2 (Ser217/221) (41G9) Rabbit mAb
(Cell Signaling Technology Cat# 9154, RRID:AB_2138017)

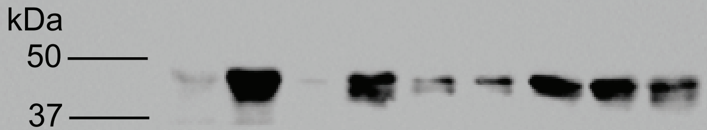

Figure 3D
MEK1/2 (D1A5) Rabbit mAb
(Cell Signaling Technology Cat# 8727, RRID:AB_10829473)

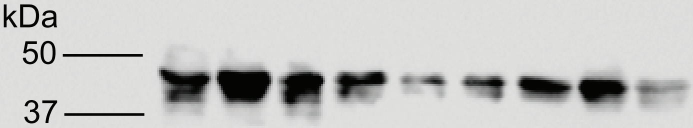

Figure 3D

Bcl-2 (D55G8) Rabbit mAb #4223

(Cell Signaling Technology Cat# 4223, RRID:AB_1903909)

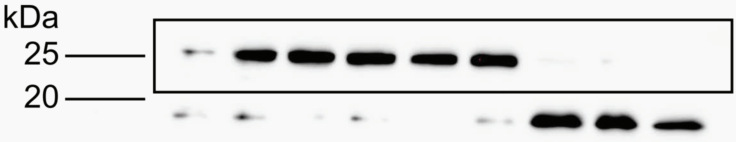

Figure 3D
Bcl-2 (D55G8) Rabbit mAb #4223
(Cell Signaling Technology Cat# 4223, RRID:AB_1903909)

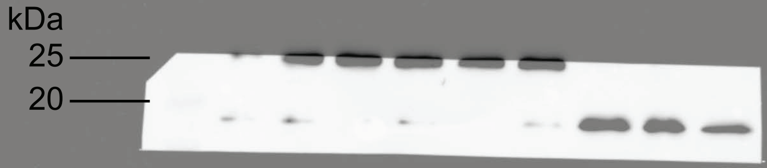

Figure 3D
GAPDH Antibody (G-9)
(Santa Cruz Biotechnology Cat# sc-365062, RRID:AB_10847862)

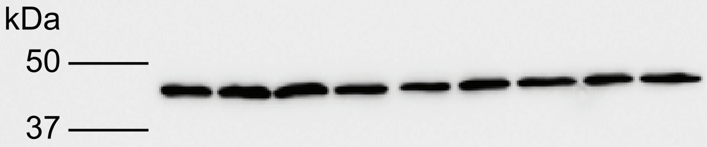

Figure 3E
Phospho-p44/42 MAPK (Erk1/2) (Thr202/Tyr204) (E10) Mouse mAb
(Cell Signaling Technology Cat# 9106, RRID:AB_331768)

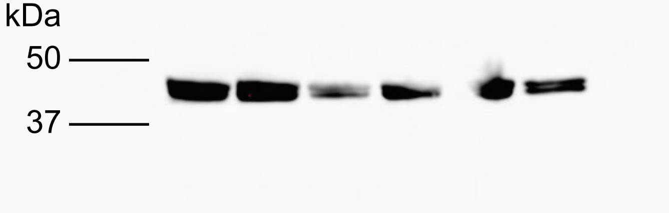

Figure 3E
p44/42 MAPK (Erk1/2) (137F5) Rabbit mAb
(Cell Signaling Technology Cat# 4695, RRID:AB_390779)

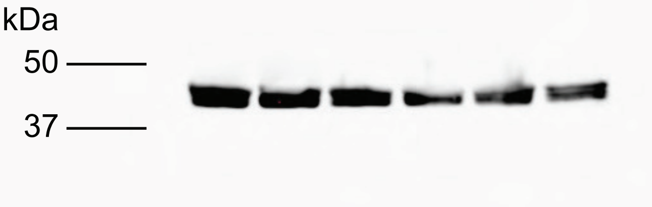

Figure 3E

Phospho-p90RSK (Ser380) (D3H11) Rabbit mAb

(Cell Signaling Technology Cat# 11989, RRID:AB_2687613)

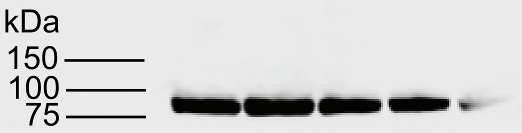

Figure 3E
RSK1 (D6D5) Rabbit mAb
(Cell Signaling Technology Cat# 8408, RRID:AB_10828594)

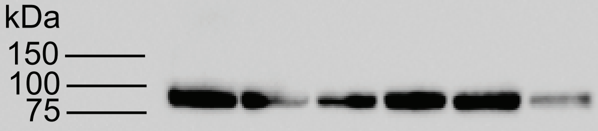

Figure 3E
Phospho-MEK1/2 (Ser217/221) (41G9) Rabbit mAb
(Cell Signaling Technology Cat# 9154, RRID:AB_2138017)

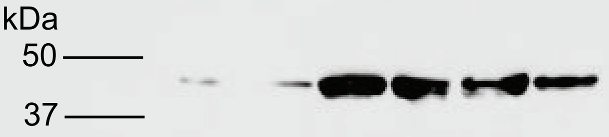

Figure 3E
MEK1/2 (D1A5) Rabbit mAb
(Cell Signaling Technology Cat# 8727, RRID:AB_10829473)

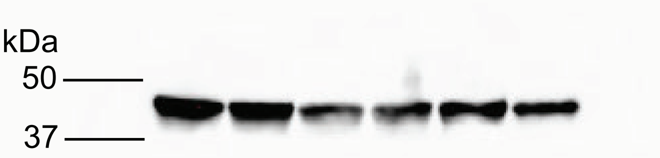

Figure 3E
Bcl-2 (D55G8) Rabbit mAb #4223
(Cell Signaling Technology Cat# 4223, RRID:AB_1903909)

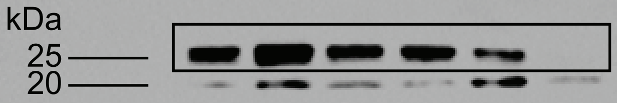

Figure 3E
GAPDH Antibody (G-9)
(Santa Cruz Biotechnology Cat# sc-365062, RRID:AB_10847862)

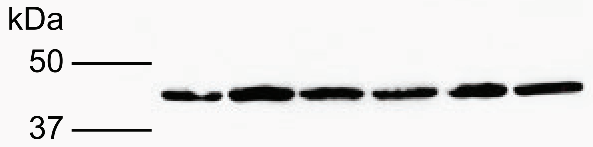

Figure 3F

Phospho-p44/42 MAPK (Erk1/2) (Thr202/Tyr204) (E10) Mouse mAb (Cell Signaling Technology Cat# 9106, RRID:AB_331768)

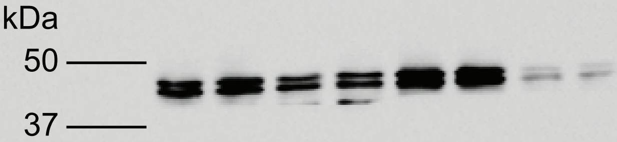

Figure 3F
p44/42 MAPK (Erk1/2) (137F5) Rabbit mAb
(Cell Signaling Technology Cat# 4695, RRID:AB_390779)

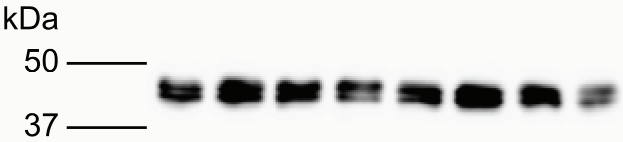

Figure 3F

Phospho-p90RSK (Ser380) (D3H11) Rabbit mAb

(Cell Signaling Technology Cat# 11989, RRID:AB_2687613)

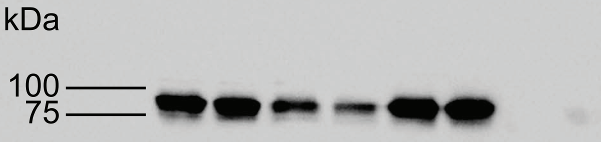

Figure 3F
Phospho-p90RSK (Ser380) (D3H11) Rabbit mAb
(Cell Signaling Technology Cat# 11989, RRID:AB_2687613)

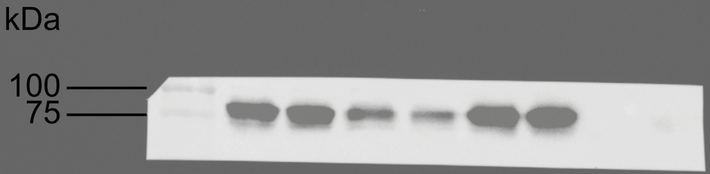

Figure 3F
RSK1 (D6D5) Rabbit mAb
(Cell Signaling Technology Cat# 8408, RRID:AB_10828594)

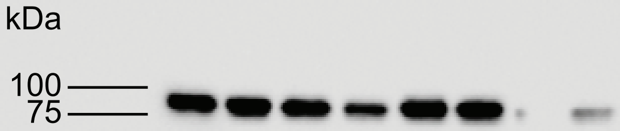

Figure 3F
Phospho-MEK1/2 (Ser217/221) (41G9) Rabbit mAb
(Cell Signaling Technology Cat# 9154, RRID:AB_2138017)

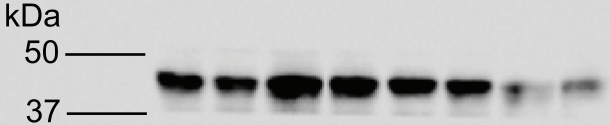

Figure 3F
MEK1/2 (D1A5) Rabbit mAb
(Cell Signaling Technology Cat# 8727, RRID:AB_10829473)

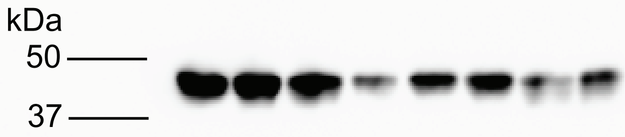

Figure 3F
GAPDH Antibody (G-9)
(Santa Cruz Biotechnology Cat# sc-365062, RRID:AB_10847862)

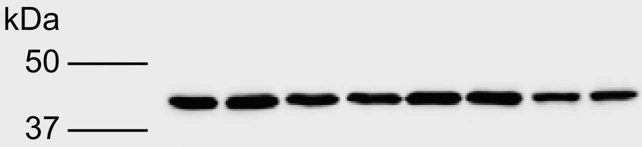

Figure 6D
Rabbit anti-CDK1 Antibody
(Thermo Fisher Scientific Cat# A303-663A, RRID:AB_11205291)

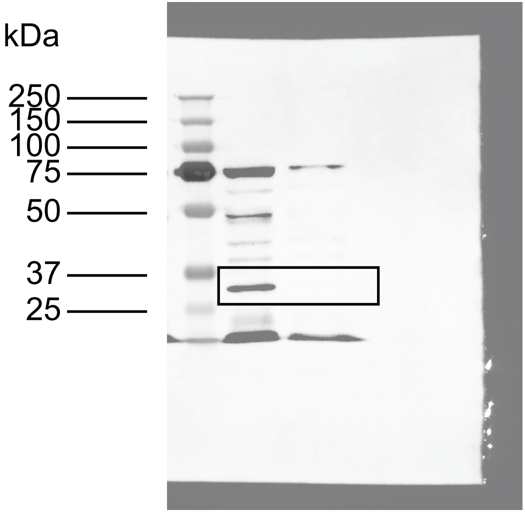

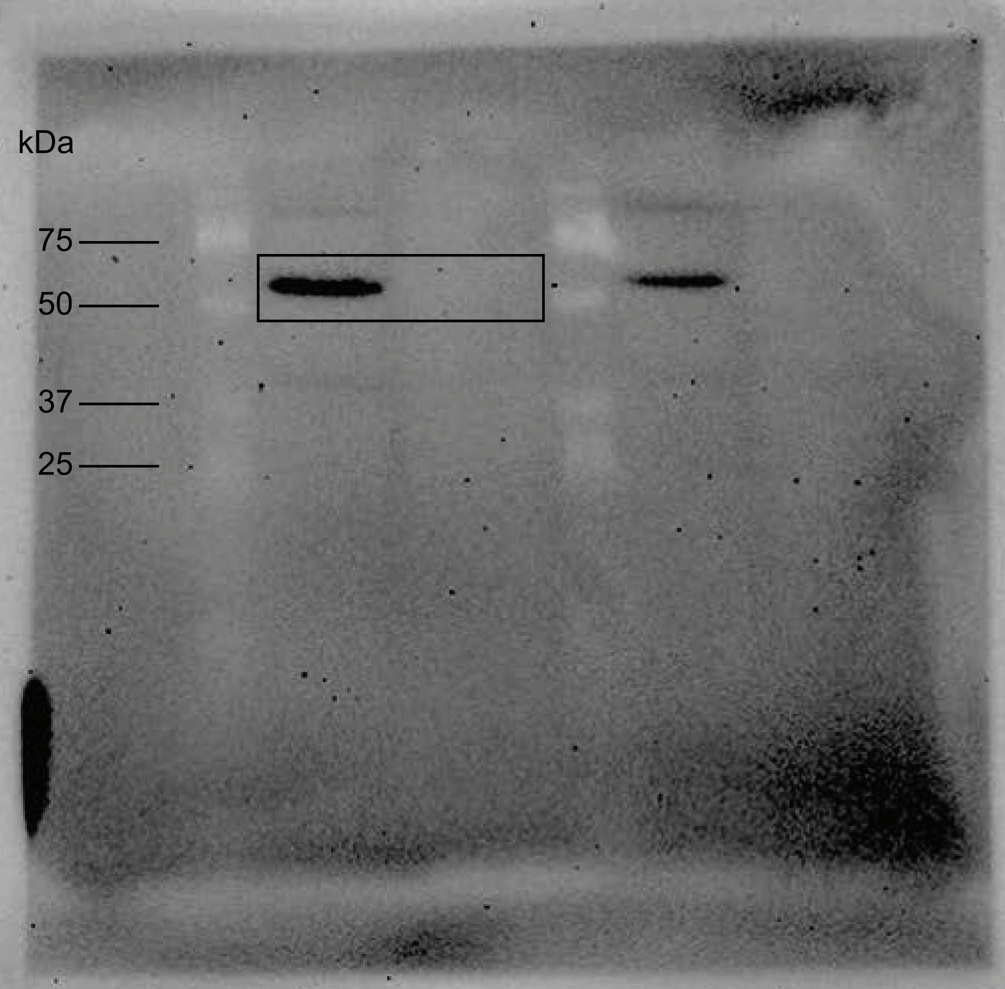

Figure 6D
CCNB1/cyclin B1 Antibody (D-11)
(Santa Cruz Biotechnology Cat# sc-7393, RRID:AB_627336)

Figure 6D
cyclin D1 Antibody (A-12)
(Santa Cruz Biotechnology Cat# sc-8396, RRID:AB_627344)

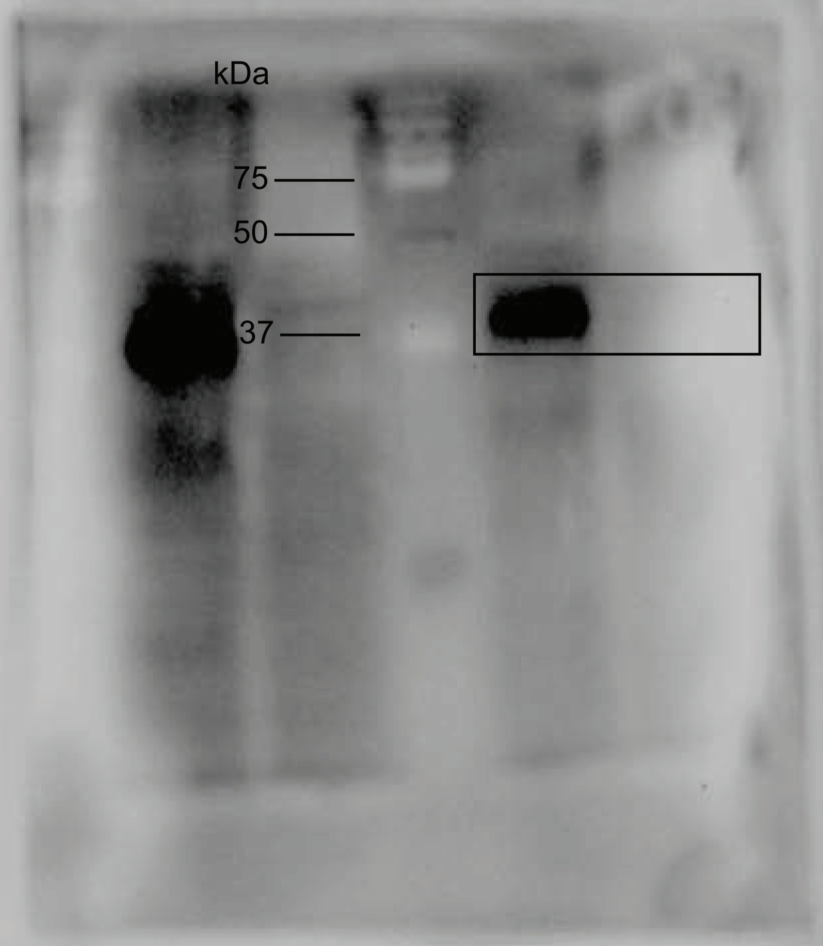

Figure 6D
CDC20 Antibody
(Cell Signaling Technology Cat# 4823, RRID:AB_10549074)

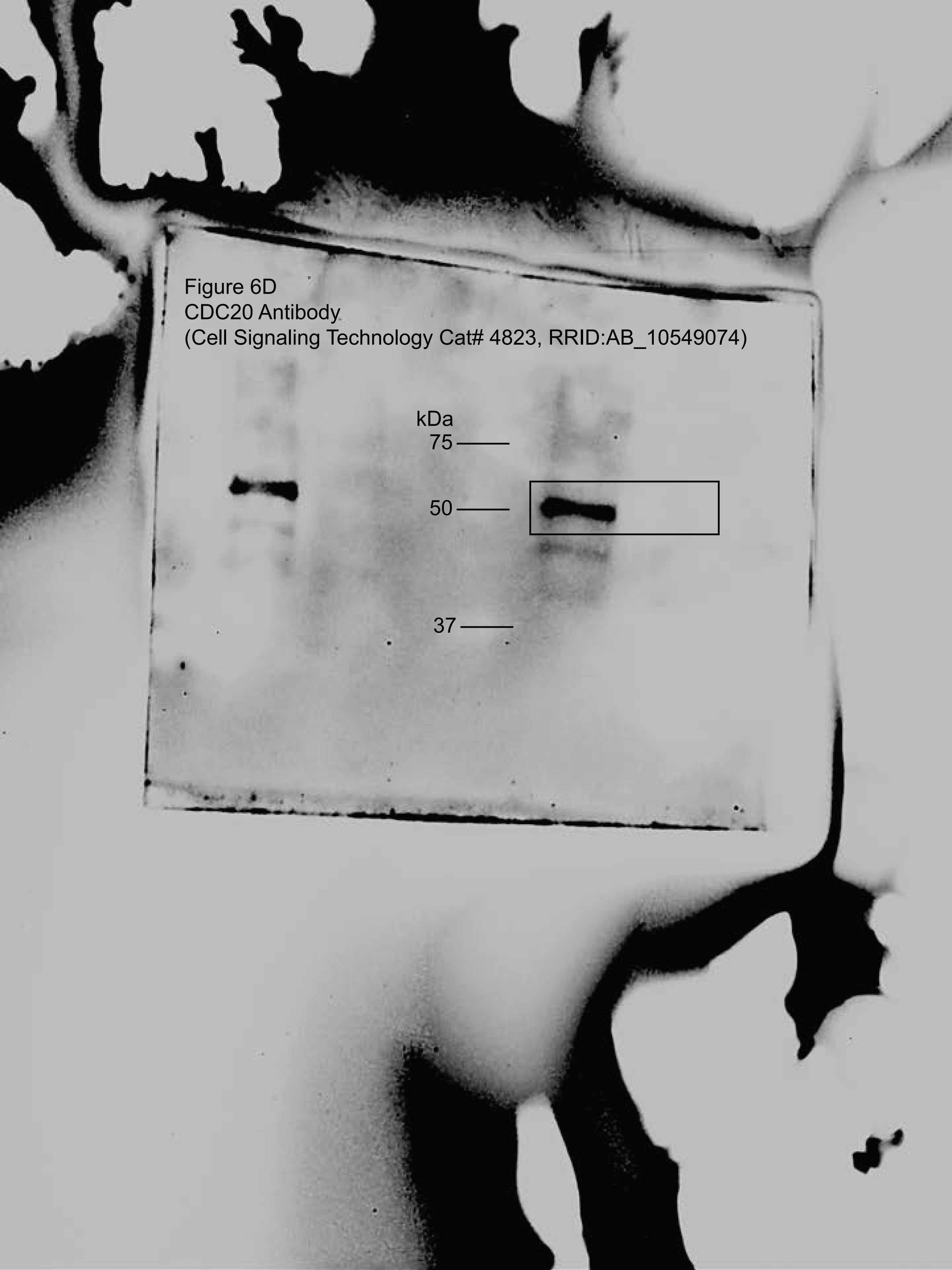

Figure 6D
GAPDH Antibody (G-9)
(Santa Cruz Biotechnology Cat# sc-365062, RRID:AB_10847862)

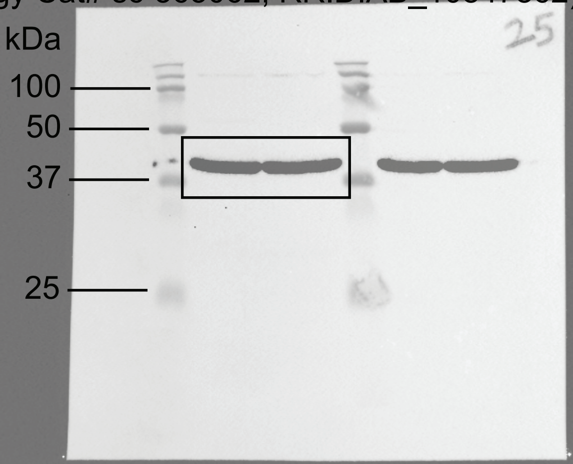

Figure S3A
Phospho-p44/42 MAPK (Erk1/2) (Thr202/Tyr204) (E10) Mouse mAb
(Cell Signaling Technology Cat# 9106, RRID:AB_331768)

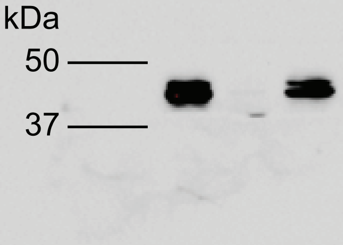

Figure S3A
p44/42 MAPK (Erk1/2) (137F5) Rabbit mAb
(Cell Signaling Technology Cat# 4695, RRID:AB_390779)

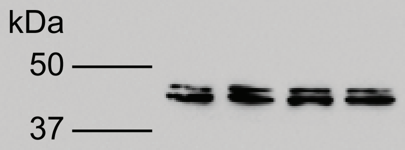

Figure S3A
Phospho-p90RSK (Ser380) (D3H11) Rabbit mAb
(Cell Signaling Technology Cat# 11989, RRID:AB_2687613)

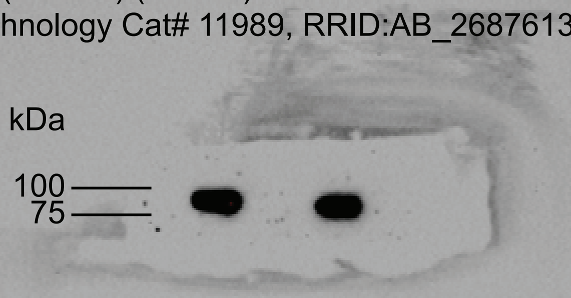

Figure S3A
RSK1 (D6D5) Rabbit mAb
(Cell Signaling Technology Cat# 8408, RRID:AB_10828594)

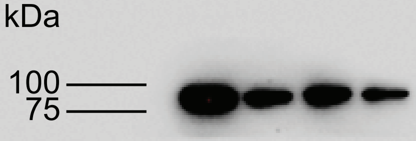

Figure S3A
Phospho-MEK1/2 (Ser217/221) (41G9) Rabbit mAb
(Cell Signaling Technology Cat# 9154, RRID:AB_2138017)

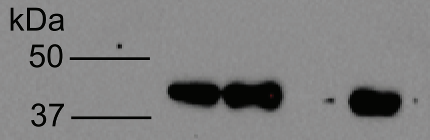

Figure S3A

MEK1/2 (D1A5) Rabbit mAb

(Cell Signaling Technology Cat# 8727, RRID:AB_10829473)

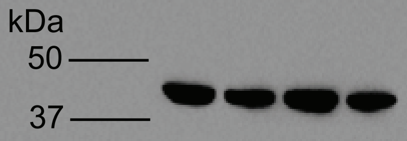

Figure S3A
GAPDH Antibody (G-9)
(Santa Cruz Biotechnology Cat# sc-365062, RRID:AB_10847862)

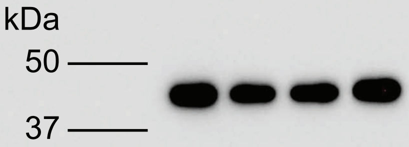

Figure S3B
Phospho-p44/42 MAPK (Erk1/2) (Thr202/Tyr204) (E10) Mouse mAb
(Cell Signaling Technology Cat# 9106, RRID:AB_331768)

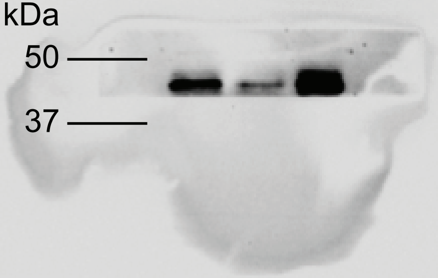

Figure S3B
p44/42 MAPK (Erk1/2) (137F5) Rabbit mAb
(Cell Signaling Technology Cat# 4695, RRID:AB_390779)

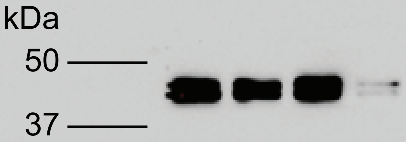

Figure S3B
Phospho-p90RSK (Ser380) (D3H11) Rabbit mAb
(Cell Signaling Technology Cat# 11989, RRID:AB_2687613)

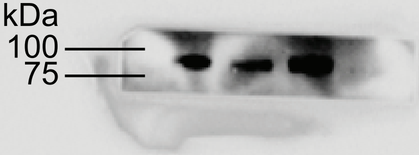

Figure S3B
RSK1 (D6D5) Rabbit mAb
(Cell Signaling Technology Cat# 8408, RRID:AB_10828594)

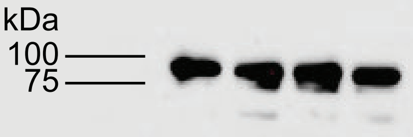

Figure S3B
Phospho-MEK1/2 (Ser217/221) (41G9) Rabbit mAb
(Cell Signaling Technology Cat# 9154, RRID:AB_2138017)

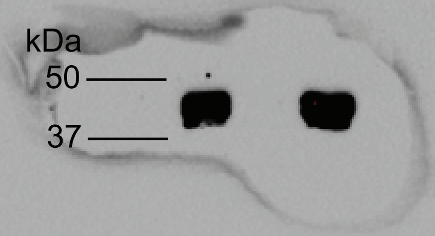

Figure S3B
MEK1/2 (D1A5) Rabbit mAb
(Cell Signaling Technology Cat# 8727, RRID:AB_10829473)

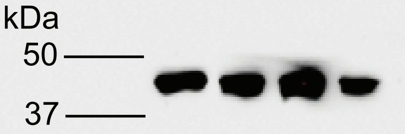

Figure S3B
GAPDH Antibody (G-9)
(Santa Cruz Biotechnology Cat# sc-365062, RRID:AB_10847862)

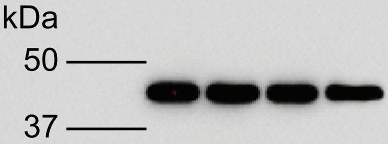

Figure S3C
Phospho-p44/42 MAPK (Erk1/2) (Thr202/Tyr204) (E10) Mouse mAb
(Cell Signaling Technology Cat# 9106, RRID:AB_331768)

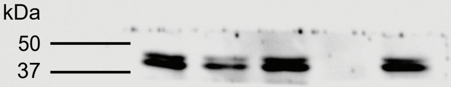

Figure S3C
p44/42 MAPK (Erk1/2) (137F5) Rabbit mAb
(Cell Signaling Technology Cat# 4695, RRID:AB_390779)

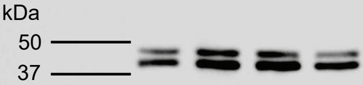

Figure S3C
Phospho-p90RSK (Ser380) (D3H11) Rabbit mAb
(Cell Signaling Technology Cat# 11989, RRID:AB_2687613)

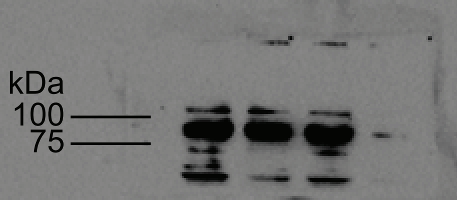

Figure S3C
RSK1 (D6D5) Rabbit mAb
(Cell Signaling Technology Cat# 8408, RRID:AB_10828594)

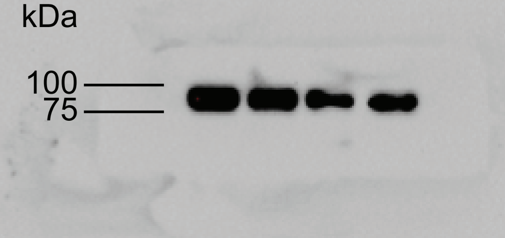

Figure S3C
Phospho-MEK1/2 (Ser217/221) (41G9) Rabbit mAb
(Cell Signaling Technology Cat# 9154, RRID:AB_2138017)

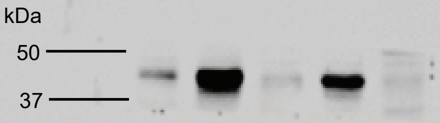

Figure S3C
MEK1/2 (D1A5) Rabbit mAb
(Cell Signaling Technology Cat# 8727, RRID:AB_10829473)

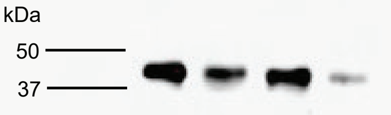

Figure S3C
GAPDH Antibody (G-9)
(Santa Cruz Biotechnology Cat# sc-365062, RRID:AB_10847862)

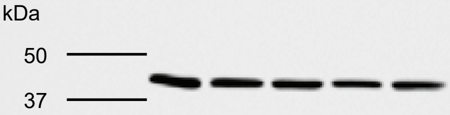

Figure S6C
Rabbit anti-CDK1 Antibody
(Thermo Fisher Scientific Cat# A303-663A, RRID:AB_11205291)

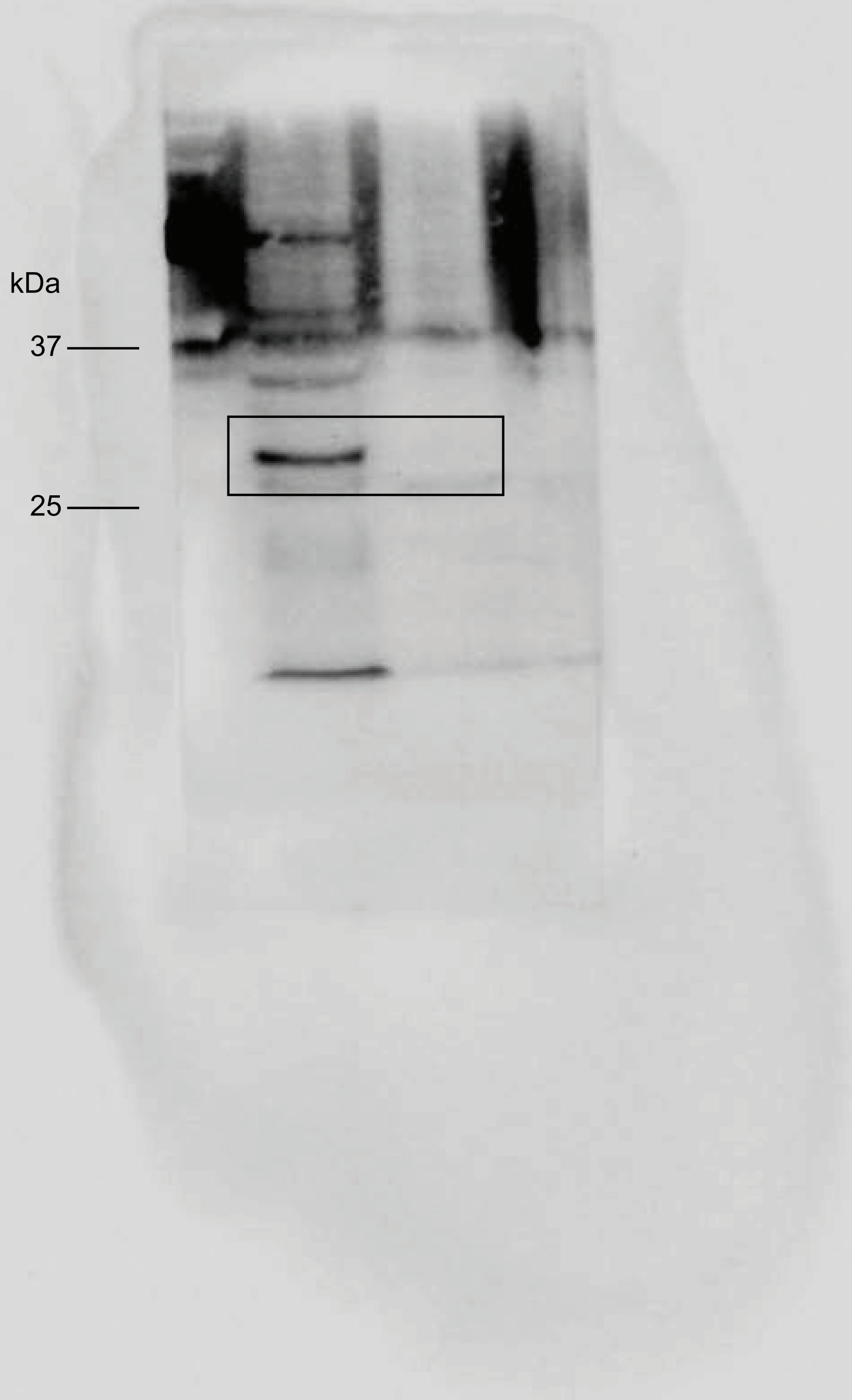

Figure S6C
CCNB1/cyclin B1 Antibody (D-11)
(Santa Cruz Biotechnology Cat# sc-7393, RRID:AB_627336)

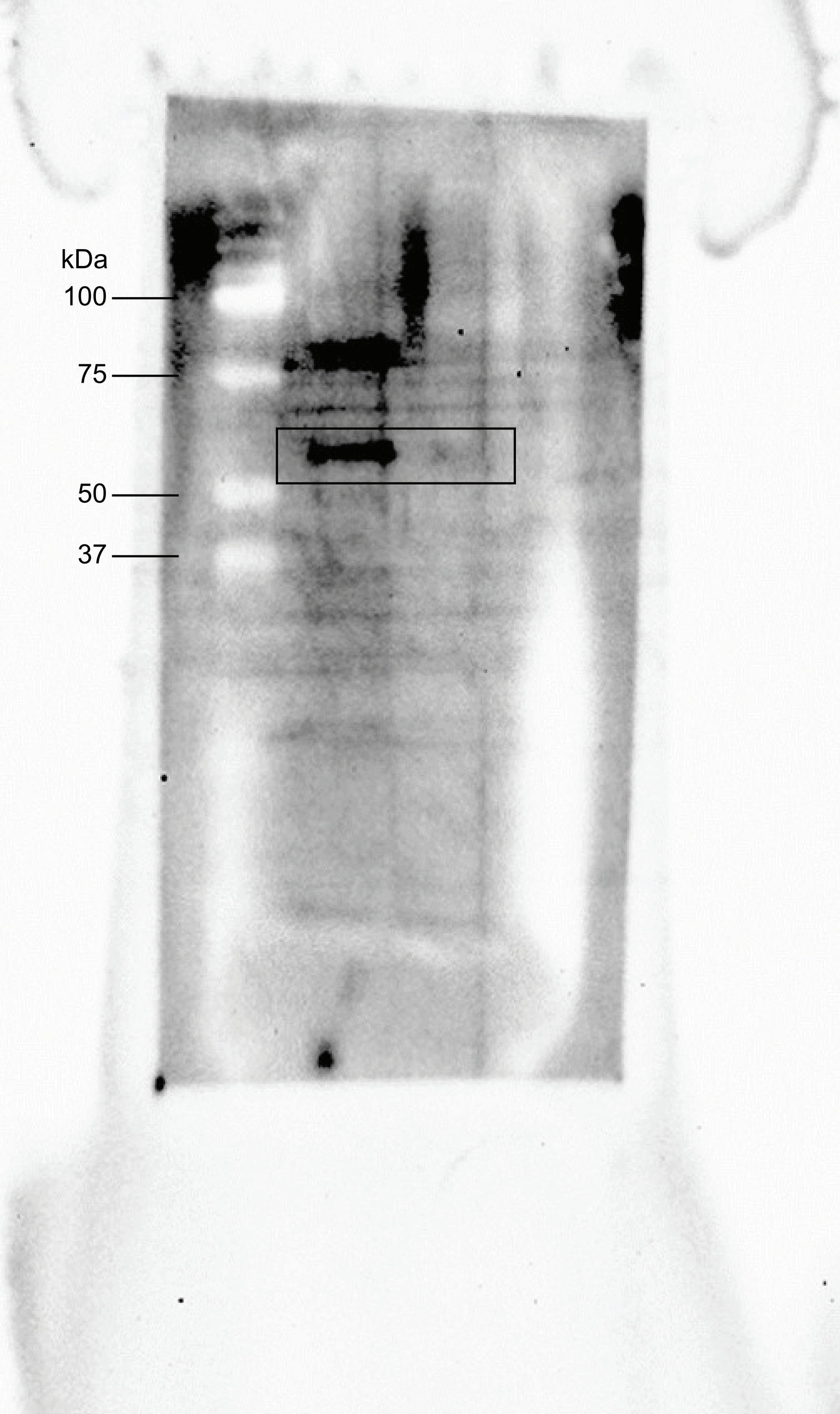

Figure S6C
cyclin D1 Antibody (A-12)
(Santa Cruz Biotechnology Cat# sc-8396, RRID:AB_627344)

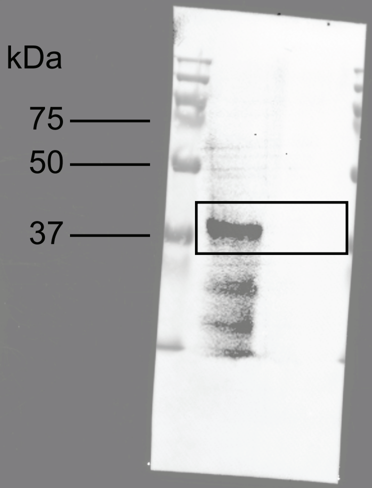

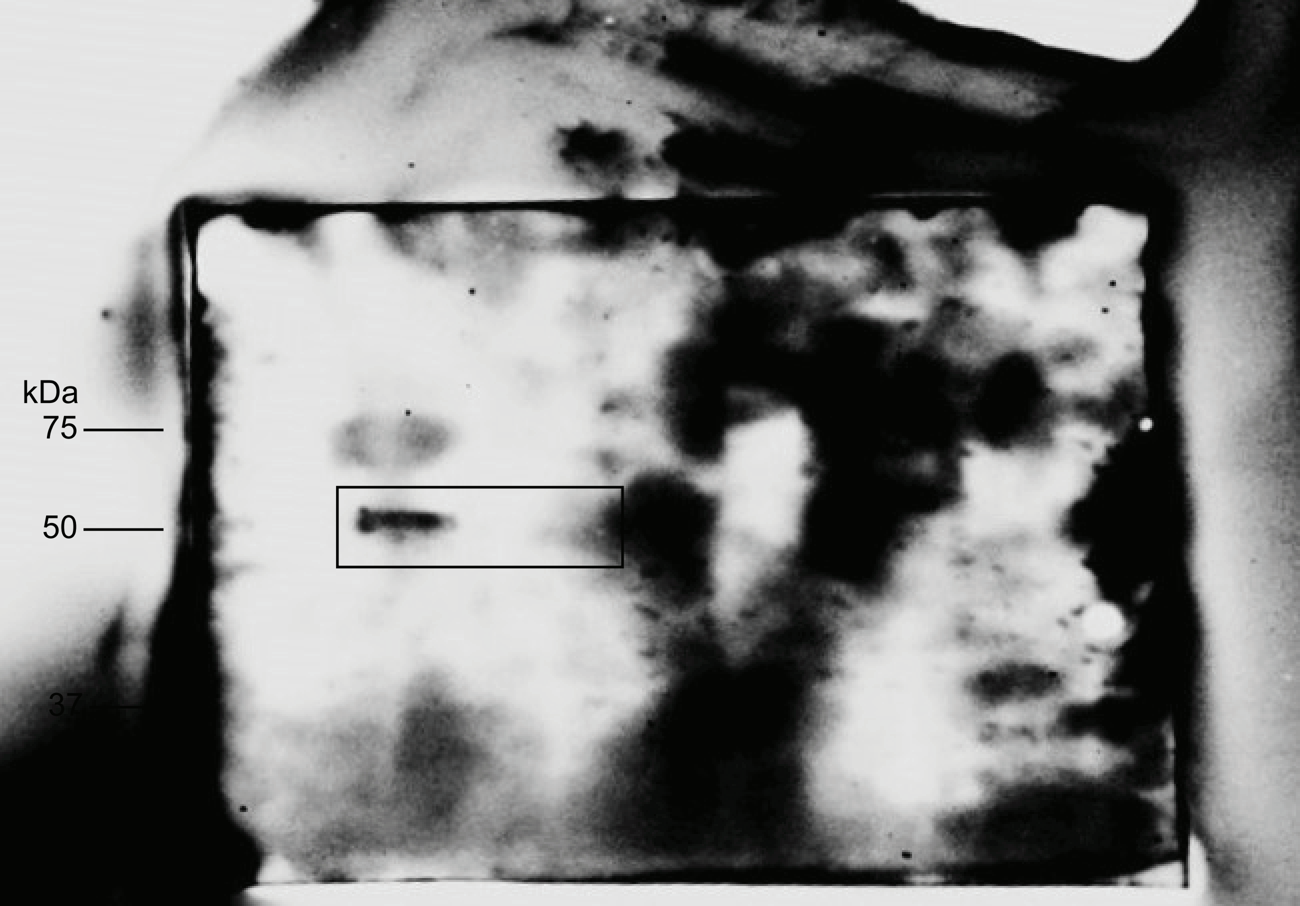

Figure S6C
CDC20 Antibody
(Cell Signaling Technology Cat# 4823, RRID:AB_10549074)

Figure S6C
GAPDH Antibody (G-9)
(Santa Cruz Biotechnology Cat# sc-365062, RRID:AB_10847862)

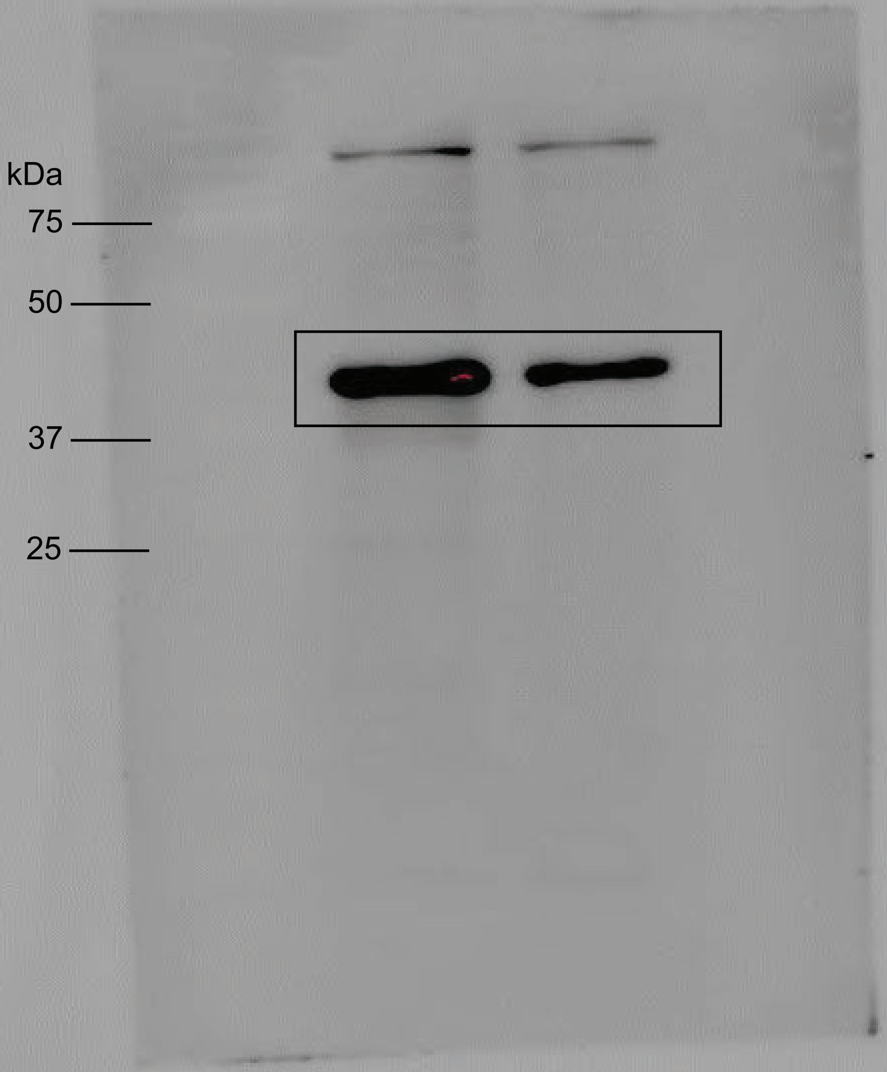

Supplement: Unedited blot and gel images [file jci-135-185220-s207.pdf]
